# Childhood trauma, adolescent risk behaviours and cardiovascular health indices in the 2004 Pelotas Birth Cohort

**DOI:** 10.1111/jcpp.14173

**Published:** 2025-04-30

**Authors:** Megan Bailey, Graeme Fairchild, Gemma Hammerton, Ina S. Santos, Luciana Tovo‐Rodrigues, Joseph Murray, Alicia Matijasevich, Sarah L. Halligan

**Affiliations:** ^1^ Department of Psychology University of Bath Bath UK; ^2^ Centre for Academic Mental Health, Population Health Sciences Bristol Medical School, University of Bristol Bristol UK; ^3^ Medical Research Council Integrative Epidemiology Unit Population Health Sciences, Bristol Medical School, University of Bristol Bristol UK; ^4^ Post‐Graduate Program in Epidemiology Federal University of Pelotas Pelotas Brazil; ^5^ Departamento de Medicina Preventiva Faculdade de Medicina FMUSP, Universidade de São Paulo São Paulo Brazil; ^6^ Department of Psychiatry and Mental Health University of Cape Town Cape Town South Africa; ^7^ Department of Psychiatry Stellenbosch University Stellenbosch South Africa

**Keywords:** Childhood trauma, adolescent, substance use, psychophysiology, risk behaviours

## Abstract

**Background:**

Childhood trauma has been associated with increased risk of substance use and poor sleep, with these factors linked to subsequent poor cardiovascular health. However, there has been little longitudinal research exploring these associations in adolescence, especially in low‐ and middle‐income countries (LMICs). To address this, we investigated longitudinal pathways from trauma to risk behaviours and cardiovascular health indices among adolescents in the 2004 Pelotas Birth Cohort, Brazil.

**Methods:**

Lifetime cumulative trauma was assessed via caregiver reports up to age 11, and combined adolescent/caregiver reports at ages 15 and 18. At age 18, current problematic alcohol use, smoking, illicit drug use and sleep duration were measured via self‐report and resting heart rate (HR) and blood pressure (BP) were assessed. We tested for trauma risk behaviour–HR/BP associations using multivariable regression, population attributable fractions and counterfactual mediation.

**Results:**

Of 4,229 adolescents (51.9% boys), 81.9% were trauma‐exposed by age 18. Cumulative trauma up to ages 15 and 18 increased the odds of age 18 alcohol, smoking and drug use (adjusted ORs: 1.25–1.44). Sleep duration was unrelated to childhood trauma. Population attributable fractions indicated that childhood trauma explained ≥28% of age 18 substance use. Unexpectedly, greater trauma exposure was associated with lower resting HR and BP. Substance use partially mediated the effect of trauma on cardiovascular health indices.

**Conclusions:**

Trauma is associated with substance use in LMIC adolescents. Prevention and intervention strategies targeting trauma are critical given this significant burden. Our finding that trauma predicts lower HR/BP warrants further exploration given well‐established associations between trauma and poorer cardiovascular health in adulthood.

## Introduction

Childhood trauma has been associated with increased odds of alcohol, smoking and drug use (and substance use disorders; Borges, Benjet, Orozco, & Medina‐Mora, 2021; Hughes et al., 2017) and with shorter sleep duration and disordered sleep (Claussen, Dimitrov, Bhupalam, Wheaton, & Danielson, 2023; Kajeepeta, Gelaye, Jackson, & Williams, 2015). Each of these factors, in turn, is linked to poor health, with childhood trauma in particular being linked to increased risk of cardiovascular diseases (CVDs; Suglia, Sapra, & Koenen, 2015), a leading cause of global morbidity (WHO, 2024). Given these observations, a potential pathway from trauma to CVD risk that operates via risk behaviours has been highlighted (Beck, Palic, Andersen, & Roenholt, 2014; Chartier, Walker, & Naimark, 2009), but the evidence base in this area has significant limitations.

First, research has focused primarily on adult samples, despite adolescence being a critical period for exposure to certain trauma types (e.g. community violence; Selner‐O'Hagan, Kindlon, Buka, Raudenbush, & Earls, 1998) and for the emergence of risk behaviours (such as substance use; Kipping, Campbell, MacArthur, Gunnell, & Hickman, 2012). Second, the majority of studies have been conducted in high‐income countries, despite greater risk of trauma exposure among youth in low‐ and middle‐income countries (LMICs; WHO, 2002). Third, the limited research that has been conducted in LMICs has predominantly adopted cross‐sectional designs, relying on retrospective reports of childhood trauma in adulthood (Kappel, Livingston, Patel, Villaveces, & Massetti, 2021), introducing potential recall biases. The only longitudinal study conducted in a LMIC that we could identify found that childhood abuse and neglect predicted alcohol and tobacco use among 9‐ to 13‐year‐olds in China (Niu, French, Wang, Sun, & Lin, 2024). However, these traumas did not predict changes in use over the 1‐year follow‐up period. There is therefore a critical need for longitudinal research on the determinants of risk behaviours among LMIC youth. Finally, there is limited evidence to inform understanding of when childhood trauma begins to impact cardiovascular health. Childhood trauma exposure has been shown to lead to changes in early markers of CVD risk, such as elevations in heart rate (HR) and blood pressure (BP; Aune et al., 2017; Yang, Magnussen, Yang, Bovet, & Xi, 2020), among adults (Goncalves Soares et al., 2021). However, evidence of equivalent effects among youth is limited and findings have been mixed (Gooding, Milliren, Austin, Sheridan, & McLaughlin, 2016; Pretty, O'Leary, Cairney, & Wade, 2013; Slopen, Koenen, & Kubzansky, 2014). Well‐powered studies that examine the full trauma‐risk behaviours–cardiovascular health pathway are particularly critical in terms of informing timely intervention efforts to mitigate the longer term impacts of childhood trauma.

We addressed these evidence gaps using data from the 2004 Pelotas Birth Cohort, an ongoing longitudinal population‐based study in Brazil. We investigated cross‐sectional and longitudinal associations between cumulative trauma exposure up to ages 11, 15 and 18, and problematic alcohol use, smoking, illicit drug use and sleep duration at age 18. We hypothesised that childhood trauma exposure would be associated with increased odds of alcohol, smoking and drug use, and with reduced sleep duration. Building on findings that childhood adversities account for ~34% of substance use disorders in US adolescents (McLaughlin et al., 2012), we computed population attributable fractions to estimate the proportion of adolescent substance use behaviours that are explained by childhood trauma exposure. Finally, we examined substance use and sleep duration as potential mediators of any effects of childhood trauma on cardiovascular health indicators of resting HR and BP. We hypothesised that trauma exposure would be associated with higher HR and BP, with risk behaviours mediating any effects.

## Methods

### Study design and population

The 2004 Pelotas Birth Cohort Study recruited children born in 2004 to mothers residing in Pelotas, Brazil (Tovo‐Rodrigues et al., 2023). All hospitals with maternity wards were visited daily, and all live births were eligible for enrolment. Children were assessed at birth, 3, 12, 24 and 48 months, then at 6, 11, 15 and 18 years. Retention was ~90% for all follow‐ups up to age 11, ~50% at age 15 (COVID‐19 pandemic interrupted data collection) and ~80% at age 18. Assessments were approved by research ethics boards at the Federal University of Pelotas and the University of São Paulo, and cohort participants/caregivers provided written informed assent/consent as appropriate. For further study details see Appendix S1 (p. 2).

### Measures

#### Childhood trauma

Child lifetime trauma exposure was assessed using the following: the post‐traumatic stress disorder subsection of the parent‐report Development and Well‐being Assessment (DAWBA; Goodman, Ford, Richards, Gatward, & Meltzer, 2000) at ages 6, 11 and 15, questionnaire items completed by adolescents at age 15 and adolescent reports on the Mini‐International Neuropsychiatric Interview (MINI; Sheehan et al., 1998) and questionnaire items at age 18 (for further details see Appendix S1, p. 3). Exposure to 12 trauma types was assessed: serious accident, fire, other disaster, attack/threat, physical abuse, sexual abuse, witnessed domestic violence, witnessed attack, witnessed accident, heard about attack, heard about accident and parental death. Cumulative trauma variables at ages 11, 15 and 18 were derived from all available data up to that timepoint, using the number of different event types reported as a proxy for *cumulative trauma load*. The resultant variables were coded as 0, 1, 2 and ≥3 exposures. Likelihood ratio tests confirmed linearity in trauma‐outcome associations and the suitability of treating cumulative trauma as a numeric variable (see Appendix S1, p. 3).

#### Adolescent risk behaviours

Risk behaviours were self‐reported by adolescents at age 18. Problematic alcohol use was assessed using the Alcohol Use Disorders Identification Test (AUDIT; Saunders, Aasland, Babor, De La Fuente, & Grant, 1993); AUDIT scores of ≥8 were coded as problematic use (Lima et al., 2005). Current smoking status and illicit drug use (cannabis, cocaine, heroin, ecstasy, amphetamines, inhalants, hallucinogens, opiates and other illicit drugs) were coded as present versus absent. Average sleep duration was assessed using the Pittsburgh Sleep Quality Index (Buysse, Reynolds, Monk, Berman, & Kupfer, 1989), previously validated for use with Brazilian adolescents (Passos et al., 2017). Sleep duration was calculated in hours as the period between going to bed and waking up minus sleep latency time. Adolescents reporting <3 hr or >13 hr of sleep were excluded (see Appendix S1, pp. 3–4).

#### Cardiovascular health indices

Resting HR and BP at age 18 were assessed after a 5‐min seated rest period using a digital automatic OMRON sphygmomanometer (model HEM‐742; OMRON Corporation; Kyoto, Japan). Two measurements were obtained with an interval of 2 min, and average resting HR, systolic BP and diastolic BP were calculated.

#### Confounders

Baseline confounders were based on maternal reports at birth and included child sex (male/female), maternal smoking and alcohol consumption during pregnancy (yes/no to any use), years of maternal education, monthly family income (in Brazilian Reals) and the day of the year that the adolescent was born (from 1 to 365; proxy for cohort birth order as this affected the age 15 follow‐up due to COVID‐19 restrictions). Child ethnicity was self‐reported at age 11 (White/other). Baseline confounders were assumed to confound all paths in our mediation models. Two intermediate confounders (potential confounders of the mediator–outcome association that could also be consequences of the exposure) were additionally accounted for in our mediation models: child BMI (age‐adjusted *z*‐scores) and physical activity (hours per week) at age 15. See Appendix S1 (p 4) for full confounder details.

### Data analysis

We pre‐registered our analyses on the Open Science Framework (https://doi.org/10.17605/OSF.IO/BG4EH; see Appendix S1, pp. 4–5), with analyses conducted using Stata Version 17. Multivariate imputation by chained equations with 50 imputed datasets was used to address missing data (for further details see Appendix S1, pp. 5–6). Findings presented here are based on imputed data (for complete case analyses see Appendix S2, pp. 9–17).

First, imputed data for the full analysis sample (*n* = 4,229) and logistic and linear regression analyses were used to examine cross‐sectional and longitudinal associations between cumulative trauma up to ages 11, 15 and 18 and problematic alcohol use, smoking, illicit drug use and sleep duration at age 18. In sensitivity analyses, we examined whether associations differed by sex. Population attributable fractions were calculated for substance use behaviours using the formula PAF = *p*_c_*(1−(1/RR)) in which p_c_ is the prevalence of trauma exposure (binary: exposed/unexposed) among those with substance use and RR is the adjusted risk ratio (Rockhill, Newman, & Weinberg, 1998). All analyses were conducted twice: first unadjusted and then adjusted for baseline confounders.

Next, due to an absence of feasible auxiliary variables for the imputation of resting HR, our mediation analyses used imputed data for a subsample who had complete resting HR data (*n* = 3,196). We used linear regression analyses to examine associations between risk behaviours and resting HR, systolic BP and diastolic BP at age 18 (mediator–outcome), unadjusted and adjusted for baseline confounders, intermediate confounders and cumulative trauma up to age 15 in a single model. Next, we performed counterfactual mediation using the parametric g‐computation formula to estimate the indirect effects of cumulative trauma up to age 15 on resting HR, systolic BP and diastolic BP at age 18 via adolescent risk behaviours. We used the – g‐formula – package in Stata to estimate the total causal effect, pure natural direct effect, and a single total natural indirect effect for all mediators simultaneously, as we did not consider the risk behaviours to be independent of each other (Daniel, De Stavola, & Cousens, 2011; De Stavola, Daniel, Ploubidis, & Micali, 2015). We performed unadjusted models and models adjusting for all baseline and intermediate confounders. Standard errors were estimated using 50 bootstrap samples and normal‐based 95% confidence intervals were calculated. We used a Monte Carlo sample size of 10,000 to minimise fluctuations in effect estimates and a set seed of 79. In sensitivity analyses, we conducted mediation analyses for each mediator individually to: (a) ensure there was no inconsistent mediation (i.e. where direct and one or more indirect effects are in opposing directions, potentially cancelling each other out in the total effects), and (b) establish whether indirect effects were being driven by one mediator in particular. These findings should be interpreted with caution, given that the potential interdependence among risk behaviours is unaccounted for in these analyses. Finally, it is important to acknowledge that mediation analyses require several assumptions to be made (De Stavola et al., 2015), including the assumption of no unmeasured confounders; the extent to which these assumptions are met determines the extent to which we can interpret our results as providing evidence for causality.

## Results

Of 4,263 live births, 4,231 infants (99.2%) were recruited into the cohort, and 4,229 adolescents (2,195 boys [51.9%] and 2034 girls [48.1%]) were included in the imputed trauma‐risk behaviour analyses. Sample characteristics according to dichotomous trauma exposure status at age 18 are presented in Table 1. By age 11, 21.7%, 7.2% and 5.3% of the cohort had been exposed to 1, 2 or ≥3 traumas in their lifetime, respectively. By age 15, 30.1%, 20.8% and 27.2% of adolescents had been exposed to 1, 2 or ≥3 traumas in their lifetime, respectively. By age 18, 36.2%, 19.8% and 25.3% of adolescents had been exposed to 1, 2 or ≥3 traumas in their lifetime, respectively. At age 18, 30.3% of adolescents reported problematic alcohol use, 8.6% reported smoking, 27.1% reported illicit drug use and the average sleep duration was 7 hr and 26 min.

**Table 1:** Sample characteristics according to trauma exposure status at age 18

	Total sample	Trauma at age 18		OR (95% CI) or mean difference	*p*‐Value
		Unexposed (18.9%)	Exposed (81.1%)		
Binary variables					
Child sex (female)	48.1 (0.01)	51.8 (0.02)	47.3 (0.01)	0.83 (0.70, 1.00)	.048
Child ethnicity (Black/other)^a^	31.9 (0.01)	25.1 (0.02)	33.6 (0.01)	1.51 (1.23, 1.85)	<.001
Maternal smoking (yes)^b^	27.5 (0.01)	20.4 (0.02)	29.1 (0.01)	1.60 (1.29, 1.99)	<.001
Maternal alcohol consumption (yes)^b^	3.3 (0.003)	1.7 (0.01)	3.7 (0.003)	2.25 (1.18, 4.30)	.014
Problematic alcohol use at age 18 (yes)	30.3 (0.01)	18.5 (0.02)	33.0 (0.01)	2.18 (1.75, 2.71)	<.001
Smoking at age 18 (yes)	8.6 (0.005)	3.3 (0.01)	9.8 (0.01)	3.27 (1.95, 5.49)	<.001
Illicit drug use at age 18 (yes)	27.1 (0.01)	18.8 (0.02)	29.1 (0.01)	1.78 (1.41, 2.25)	<.001
Continuous variables					
Monthly family income, BRL^c^	803.59 (17.1)	900.15 (49.3)	781.12 (19.5)	−119.03 (−224.81, −13.25)	.028
Maternal education, years	8.13 (0.05)	8.46 (0.1)	8.05 (0.1)	−0.41 (−0.73, −0.09)	.012
Cohort birth order^d^	179.88 (1.6)	201.44 (4.1)	174.87 (1.8)	−26.57 (−35.58, −17.57)	<.001
Sleep duration at age 18 (hr)	7.43 (0.02)	7.47 (0.06)	7.42 (0.03)	−0.04 (−0.17, 0.09)	.497

Based on imputed data (*N* = 4,229). Data are % (SE) for binary variables and mean (SE) for continuous variables, unless stated otherwise. BRL, Brazilian real; CI, confidence interval; OR, odds ratio.

^a^ Reference group is White.

^b^ During any trimester of pregnancy.

^c^ Conversion rate on 1 January 2004: 1 BRL = 0.34 USD.

^d^ Ranked date of birth relative to other cohort members (lower numbers correspond to birth earlier in the year).

### Effects of cumulative trauma up to ages 11, 15 and 18 on risk behaviours at age 18

We examined associations between cumulative trauma up to ages 11, 15 and 18 and problematic alcohol use, smoking, illicit drug use and sleep duration at age 18. For trauma up to age 11, each category increase in cumulative trauma was associated with increased odds of illicit drug use at age 18 (adjusted odds ratio (aOR) = 1.12 [95% CI 1.03–1.23]), but there was no evidence of associations for problematic alcohol use, smoking and sleep duration. For trauma up to age 15, each category increase in cumulative trauma was associated with increased odds of problematic alcohol use (1.30 [1.18–1.43]), smoking (1.43 [1.23–1.65]) and illicit drug use (1.25 [1.13–1.37]) at age 18, with no effects for sleep duration. Similarly, for concurrent trauma‐risk behaviour associations at age 18, each category increase in cumulative trauma was associated with increased odds of problematic alcohol use (1.38 [1.28–1.48]), smoking (1.44 [1.28–1.62]) and illicit drug use (1.27 [1.17–1.38]), but sleep duration was again unrelated to trauma (Table 2). Sensitivity analyses indicated some sex differences for substance use, such that greater odds were observed for males than females in three out of nine analyses, specifically: the associations between cumulative trauma up to ages 11 and 18 and illicit drug use at age 18; and the association between cumulative trauma up to age 15 and smoking at age 18 (see Appendix S2, p. 2).

**Table 2:** Cross‐sectional and longitudinal associations between cumulative trauma up to ages 11, 15 and 18 and adolescent risk behaviours at age 18

	Cumulative trauma up to age 11		Cumulative trauma up to age 15		Cumulative trauma up to age 18	
	Coefficient (95% CI)	*p*‐Value	Coefficient (95% CI)	*p*‐Value	Coefficient (95% CI)	*p*‐Value
Unadjusted						
Problematic alcohol use^a^	1.06 (0.96, 1.16)	.238	1.31 (1.20, 1.45)	<.001	1.40 (1.30, 1.51)	<.001
Smoking	1.19 (1.03, 1.37)	.017	1.48 (1.29, 1.70)	<.001	1.53 (1.37, 1.71)	<.001
Illicit drug use	1.16 (1.06, 1.26)	.001	1.26 (1.15, 1.39)	<.001	1.31 (1.21, 1.41)	<.001
Sleep duration (hr)	0.002 (−0.06, 0.07)	.956	−0.03 (−0.08, 0.03)	.331	−0.01 (−0.06, 0.04)	.770
Adjusted						
Problematic alcohol use^a^	1.03 (0.93, 1.13)	.600	1.30 (1.18, 1.43)	<.001	1.38 (1.28, 1.48)	<.001
Smoking	1.12 (0.97, 1.29)	.122	1.43 (1.23, 1.65)	<.001	1.44 (1.28, 1.62)	<.001
Illicit drug use	1.12 (1.03, 1.23)	.011	1.25 (1.13, 1.37)	<.001	1.27 (1.17, 1.38)	<.001
Sleep duration (hr)	−0.01 (−0.08, 0.05)	.753	−0.03 (−0.09, 0.02)	.278	−0.03 (−0.08, 0.02)	.318

Based on imputed data (*N* = 4,229). Coefficients for binary risk behaviours (problematic alcohol use, smoking and illicit drug use) are odds ratios. Coefficients for continuous risk behaviours (sleep duration) are unstandardised beta. Confounders include child sex, child ethnicity, maternal smoking during pregnancy, maternal alcohol consumption during pregnancy, maternal education at birth, monthly family income at birth and cohort birth order. CI, confidence interval.

^a^ Based on the Alcohol Use Disorders Identification Test (AUDIT); adolescents scoring 8 and above were coded as having problematic alcohol use.

Population attributable fraction estimates showed that trauma exposure up to age 18 (binary: exposed/unexposed) accounted for 36.8% (95% CI 26.9–45.1) of problematic alcohol use, 59.1% (37.3–72.3) of smoking and 27.9% (15.7–38.1) of illicit drug use at age 18 (for the analyses underpinning these results see Appendix S2, Table S1, p. 4).

### Associations between risk behaviours and cardiovascular health indices at age 18

Of the 4,231 individuals recruited into the cohort, 3,196 adolescents (75.5% of the original cohort; 1,617 boys [50.6%] and 1,579 girls [49.4%]) were included in the imputed mediator–outcome regression and mediation analyses. In adjusted models, problematic alcohol use at age 18 was concurrently associated with lower resting HR (*B* = −2.18 [95% CI −3.29, −1.07]) and diastolic BP (−0.94 [−1.59, −0.29], *p* = .005), but not systolic BP. Furthermore, age 18 smoking was concurrently associated with lower resting systolic (−2.81 [−4.37, −1.24]) and diastolic BP (−2.00 [−3.08, −0.91]), but not HR. We found no evidence of associations between age 18 illicit drug use or sleep duration and any cardiovascular health indices (Table 3).

**Table 3:** Cross‐sectional associations between adolescent risk behaviours at age 18 and resting HR, systolic BP and diastolic BP at age 18

	Problematic alcohol use		Smoking		Illicit drug use		Sleep duration	
	*B* (95% CI)	*p*	*B* (95% CI)	*p*	*B* (95% CI)	*p*	*B* (95% CI)	*p*
Unadjusted								
HR	−3.15 (−4.28, −2.03)	<.001	−0.69 (−2.62, 1.23)	.482	−1.53 (−2.69, −0.37)	.010	0.30 (−0.09, 0.69)	.130
Systolic BP	−0.50 (−1.58, 0.58)	.364	−2.00 (−3.77, −0.23)	.027	−0.32 (−1.41, 0.77)	.564	−0.67 (−1.02, −0.31)	<.001
Diastolic BP	−1.33 (−1.99, −0.68)	<.001	−1.69 (−2.78, −0.60)	.002	−0.56 (−1.23, 0.11)	.100	−0.08 (−0.30, 0.15)	.502
Adjusted								
HR	−2.18 (−3.29, −1.07)	<.001	−0.14 (−2.05, 1.77)	.886	−0.64 (−1.78, 0.51)	.275	−0.10 (−0.48, 0.28)	.613
Systolic BP	−0.62 (−1.57, 0.33)	.201	−2.81 (−4.37, −1.24)	<.001	−0.33 (−1.29, 0.63)	.496	0.01 (−0.30, 0.32)	.945
Diastolic BP	−0.94 (−1.59, −0.29)	.005	−2.00 (−3.08, −0.91)	<.001	−0.28 (−0.93, 0.38)	.413	−0.14 (−0.36, 0.08)	.217

Based on imputed data (*n* = 3,196). Confounders include child sex, child ethnicity, maternal smoking during pregnancy, maternal alcohol consumption during pregnancy, maternal education at birth, monthly family income at birth, cohort birth order, cumulative trauma up to age 15, age‐adjusted BMI and estimated hours of physical activity at age 15. BP, blood pressure; CI, confidence interval; HR, heart rate.

### Mediation effects of cumulative trauma up to age 15 on cardiovascular health indices via substance use behaviours

**Figure 1:**
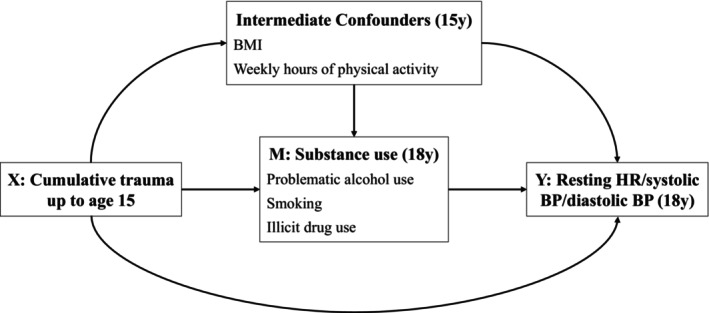
Directed acyclic graph (DAG) showing hypothesised causal pathways between cumulative trauma up to age 15 and resting HR, systolic BP and diastolic BP at age 18 through mediating substance use behaviours. Baseline confounders (not shown on DAG but assumed to confound all paths) included child sex, child ethnicity, maternal smoking during pregnancy, maternal alcohol consumption during pregnancy, maternal education at birth, monthly family income at birth and cohort birth order. BP, blood pressure; HR, heart rate

Sleep duration at age 18 was unrelated to trauma and cardiovascular health indices and was therefore excluded from mediation models. Problematic alcohol use, smoking, and illicit drug use were examined as potential mediators of the effects of cumulative trauma on the cardiovascular health indices. The directed acyclic graph for our final model is presented in Figure 1. Table 4 shows the total causal effect, natural direct effect and natural indirect effect of cumulative trauma up to age 15 on resting HR, systolic BP, and diastolic BP at age 18 via all three mediators simultaneously. After adjusting for baseline and intermediate confounders, there was evidence of a total effect of cumulative trauma up to age 15 on lower resting HR (*B* = −1.26 [95% CI −1.88, −0.65]), systolic BP (−0.61 [−1.13, −0.08]) and diastolic BP (−0.72 [−1.06, −0.38]). The effect was strongest for HR, where adolescents exposed to ≥3 traumas up to age 15 had an average resting HR that was ~4 bpm lower at age 18 compared to unexposed adolescents (78.69 bpm vs. 82.95 bpm; see Appendix S2, Table S2, p. 5).

**Table 4:** Mediation models of the effect of cumulative trauma up to age 15 on resting HR, systolic BP and diastolic BP at age 18 through mediating substance use behaviours (problematic alcohol use, smoking and illicit drug use; simultaneously included in the models)

	Unadjusted	Adjusted
Resting HR		
Total causal effect	−1.30 (−1.93, −0.68)	−1.26 (−1.88, −0.65)
Natural direct effect	−1.17 (−1.81, −0.53)	−1.15 (−1.77, −0.54)
Natural indirect effect	−0.14 (−0.25, −0.03)	−0.11 (−0.20, −0.03)
Proportion mediated	10.5%	8.7%
Resting systolic BP		
Total causal effect	−0.42 (−1.02, 0.17)	−0.61 (−1.13, −0.08)
Natural direct effect	−0.36 (−0.97, 0.25)	−0.53 (−1.05, −0.01)
Natural indirect effect	−0.06 (−0.15, 0.03)	−0.07 (−0.15, 0.01)
Proportion mediated	14.8%	12.2%
Resting diastolic BP		
Total causal effect	−0.69 (−1.01, −0.37)	−0.72 (−1.06, −0.38)
Natural direct effect	−0.60 (−0.94. −0.27)	−0.63 (−0.98, −0.29)
Natural indirect effect	−0.09 (−0.14, −0.03)	−0.09 (−0.13, −0.04)
Proportion mediated	12.6%	11.9%

Based on imputed data (*n* = 3,196). Baseline confounders include child sex, child ethnicity, maternal smoking during pregnancy, maternal alcohol consumption during pregnancy, maternal education at birth, monthly family income at birth and cohort birth order. Intermediate confounders include age‐adjusted BMI and estimated hours of physical activity at age 15. BP, blood pressure; HR, heart rate.

In terms of mediation, there was evidence of a natural indirect effect of cumulative trauma up to age 15 on age 18 HR via the three substance use behaviours (*B* = −0.11 [95% CI −0.20, −0.03]), with the indirect effect comprising 8.7% of the total effect. A similar indirect effect was identified for diastolic BP (−0.09 [−0.13, −0.04]), comprising 11.9% of the total effect. Evidence for the natural indirect effect of cumulative trauma up to age 15 on resting systolic BP at age 18 via the mediators was weaker (−0.07 [−0.15, 0.01]), with 95% confidence intervals for the estimated effect crossing zero.

Finally, we conducted sensitivity analyses to explore whether the indirect effects of cumulative trauma on cardiovascular health indices via the substance use behaviours were potentially driven by a single mediator. Nine single models tested each of the three risk behaviours as potential mediators against HR, systolic BP and diastolic BP, adjusting for baseline and intermediate confounders. We found evidence that the effect of cumulative trauma on HR was primarily mediated by problematic alcohol use (proportion mediation = 7.6%); the effect of cumulative trauma on systolic BP was primarily mediated by smoking (proportion mediation = 16.4%); and the effect of cumulative trauma on diastolic BP was driven by both problematic alcohol use and smoking (proportion mediation = 6.2% and 7.5%, respectively). There was no evidence for any indirect effects via illicit drug use. Full details of these results are presented in Appendix S2 (Tables S3–S5, pp. 6–8).

## Discussion

In this large Brazilian birth cohort, we observed both concurrent and longitudinal associations between cumulative trauma exposure and problematic alcohol use, smoking and illicit drug use at age 18, but no associations between trauma and sleep duration. Population attributable fractions estimated that trauma exposure accounted for ≥28% of substance use behaviours at age 18, with particularly strong effects for smoking. We also identified associations between cumulative trauma and cardiovascular health indices at age 18, with evidence that these effects were partially mediated by substance use. Unexpectedly, cumulative trauma and substance use behaviours were associated with lower, rather than higher, resting HR and BP.

We found evidence of a persistent effect of cumulative childhood trauma on substance use behaviours, including evidence of a ‘dose‐dependent’ relationship between cumulative trauma up to ages 15 and 18 and problematic alcohol use, smoking, and illicit drug use at age 18. Previous research has demonstrated associations between childhood trauma and these behaviours among adolescents in high‐income countries (Amorim, Soares, Abrahamyan, Severo, & Fraga, 2023). Our study provides some of the first longitudinal evidence of these associations in adolescents living in LMICs. Our observations that childhood trauma is linked to substance use in this non‐clinical, population‐representative sample highlight the potential importance of traumatic experiences for engagement in substance use for a significant proportion of LMIC youth. We also found evidence that the association between trauma exposure and substance use behaviours may be stronger in males than females, though further investigation is warranted given that we only observed this in three out of nine analyses.

While we found robust effects of cumulative trauma exposure at ages 15 and 18 on adolescent substance use behaviours, findings were less consistent for trauma up to age 11, which was only predictive of later illicit drug use. It is possible that more proximal trauma exposures are more influential in shaping adolescent risk behaviours, or that adolescents versus children are more likely to experience trauma types (e.g. community violence; Selner‐O'Hagan et al., 1998) that are strongly linked to substance abuse (Lee, 2012). There were also changes in how trauma was assessed over time in the cohort; age 11 trauma was based only on maternal reports, whereas at ages 15 and 18 maternal and adolescent reports were combined. It may be that some mothers were unaware of the full range of their children's traumatic experiences at age 11. Additionally, as adolescents reported on their own risk behaviours at age 18, it is also possible that informant bias magnified associations when trauma indices also included adolescent reports. Consistent with this, previous research has highlighted that retrospective self‐reports of childhood maltreatment may be more strongly associated with psychopathology than prospective informant reports (Newbury et al., 2018).

Surprisingly, we found that cumulative trauma was associated with *lower* resting HR and BP at age 18. Substance use behaviours were also negatively associated with these cardiovascular health indices: problematic alcohol use was associated with lower resting HR and diastolic BP, and smoking with lower systolic and diastolic BP. Moreover, in combination, alcohol, smoking and illicit drug use partially mediated the effect of cumulative trauma on these cardiovascular health indices, though effects were small, with preliminary evidence indicating that problematic alcohol use and smoking were important individual contributors to these indirect effects.

Given that both childhood trauma and substance use are linked to poorer cardiovascular health in adulthood (WHO, 2011), our observations that each of these domains was associated with *lower* HR and BP were counter to our predictions. Previous research investigating the association between childhood trauma and cardiovascular health indices in youth has yielded mixed findings, with positive, negative and null associations reported (Gooding et al., 2016; Pretty et al., 2013; Slopen et al., 2014). Additionally, most studies have observed effects for HR but not BP, and vice versa. Effects may be more robust in adulthood (Su et al., 2015), though the evidence base is still characterised by some mixed findings, especially for BP (Goncalves Soares et al., 2021; Schreier, Jones, Nayman, & Smyth, 2019). Similarly, existing evidence with respect to substance use has also reported mixed findings, with alcohol use and smoking linked to elevated HR and BP (Hayibor, Zhang, & Duncan, 2019; Papathanasiou et al., 2013), as well as with reduced HR and BP at light‐to‐moderate levels of use (Gémes et al., 2016; Li et al., 2017). Further exploration of these associations is warranted, particularly regarding the effects of threat‐related versus deprivation‐related adversities, which may affect the cardiovascular system in different ways (Busso, McLaughlin, & Sheridan, 2017).

Pre‐existing psychopathology may offer a possible explanation for our findings. Low resting HR has been associated with externalising problems (e.g. conduct problems and antisocial behaviour) in children and adolescents in observational studies (Ortiz & Raine, 2004), although causal effects have yet to be demonstrated using more robust methods (e.g. Mendelian Randomisation; Karwatowska et al., 2023). Externalising problems have also been associated with increased odds of trauma exposure (Carliner, Gary, McLaughlin, & Keyes, 2017), including in the current cohort at ages 6 and 11 (Bauer et al., 2022), and with alcohol and drug use among adolescents (Heradstveit et al., 2018). Thus, pre‐existing externalising psychopathology may partially explain why cumulative trauma and substance use behaviours were both associated with lower HR and BP in our sample. Future research should track trauma–mental health‐risk behaviour associations across the lifespan and investigate how these risks are related to cardiovascular health indices and subsequent cardiovascular health over time. Moreover, the pathway from childhood trauma exposure to cardiovascular health outcomes is likely to be much more complex, involving multiple, overlapping pathways, including epigenetics and biological and physiological changes. Moving forward, longitudinal and cohort studies should aim to collect detailed biological and epigenetic information alongside the assessment of psychosocial and environmental factors to enable the comprehensive investigation of the long‐term impact of childhood trauma exposure on health throughout the lifespan.

Notably, cumulative trauma was not associated with sleep duration in our LMIC adolescent sample, in contrast to previous evidence linking childhood adversities with short sleep duration (Claussen et al., 2023). Research has also reported associations between childhood trauma and longer sleep duration (Schneiderman, Ji, Susman, & Negriff, 2018), though there were no indications of this in the current sample (see Appendix S1, pp. 3–4). We also found no evidence of associations between sleep duration and cardiovascular health indices at age 18. In combination, our findings suggest that there may be no persistent consequences of childhood trauma on sleep duration, and that sleep may not be a key contributor to trauma‐cardiovascular health associations. However, future research should utilise more objective sleep indices, such as those measured through actigraphy, to further explore these associations.

Overall, our findings confirm childhood trauma as a key risk factor for substance use behaviours and suggest that these behaviours may partially mediate the effect of childhood trauma on changes in cardiovascular health indices. The long‐term association to adverse cardiovascular health is still unclear given the present study's unexpected findings that trauma‐exposed adolescents had lower HR and BP than unexposed adolescents. It is possible that the repeated exposure to trauma, or exposure during different developmental periods, may affect cardiovascular systems and stress responses differently in adolescence compared to adulthood (Herzog, D'Andrea, DePierro, & Khedari, 2018). Nevertheless, our population attributable fraction estimates suggest that childhood trauma exposure accounts for between 28% and 59% of all adolescent substance use. Future research should explore potential mechanisms underlying this association to expand our understanding of why childhood trauma leads to an increased vulnerability for trying and continuing to use substances. Prevention strategies to reduce exposure to childhood trauma as well as interventions targeting those exposed to trauma could be critical in reducing the substantial global health burden of substance use and associated disorders (Degenhardt et al., 2018).

Our study addressed several key limitations of the existing literature. We investigated the association between childhood trauma and risk behaviours during adolescence, a key period of risk for both trauma exposure and the emergence of these behaviours (Kipping et al., 2012; Selner‐O'Hagan et al., 1998). Additionally, our large and well‐characterised population‐based sample meant that trauma exposure was captured using prospective data across four timepoints and multiple informants, minimising the impact of retrospective recall and informant bias and relevant confounders were measured and adjusted for in all analyses. In addition, we examined the full pathway, from childhood trauma to cardiovascular health indices via substance use behaviours, with equivalent evidence being surprisingly scarce. However, several limitations must also be acknowledged. First, we were unable to collect detailed temporal information regarding the precise timing of trauma exposure and risk behaviour onset, therefore it is technically possible that the onset of these behaviours could have preceded trauma exposure, particularly in adolescence (Borges et al., 2021). Furthermore, the temporal specificity of our mediation analyses is limited as both substance use and cardiovascular health indices were measured at age 18, though this does improve on similar mediation analyses which were conducted solely using cross‐sectional data from older, high‐income country samples (Beck et al., 2014; Chartier et al., 2009). Second, though our mediation analyses allow us to move closer to causality, any bias in measured or unmeasured confounders, reverse causality, selection bias, or measurement error would affect our ability to make strong causal inferences. Third, we were unable to examine whether trauma exposure led to changes in HR and BP across time. Future population‐based studies should aim to collect detailed temporal information regarding trauma timing and the onset of risk behaviours, and to repeatedly assess cardiovascular health indices to enable their long‐term examination. Additionally, although simple and cost‐effective to measure at scale, HR and BP provide only limited insight into cardiovascular health (Su et al., 2015). Other indices, such as arterial stiffness (Rafiq, O'Leary, Dempster, Cairney, & Wade, 2020), could potentially be more sensitive to early indicators of cardiovascular health problems in the context of trauma. Moreover, ≥50% of the cohort had missing data at age 15 due to the COVID‐19 pandemic interrupting data collection. Finally, our findings should be interpreted in the context of Pelotas, a predominantly urban area with a higher income inequality and lower gross domestic product per capita compared to the Brazilian national average (see Appendix S1, p. 2); the generalisability of our findings may therefore be limited given the considerable socioeconomic variation in Brazil.

## Conclusion

We found both concurrent and longitudinal associations between cumulative trauma exposure and problematic alcohol use, smoking, and illicit drug use in adolescence, such that an increasing number of trauma exposures increased the odds of engaging in these behaviours at age 18. We found that both cumulative trauma and substance use were associated with lower resting HR and systolic and diastolic BP, necessitating further investigation of these factors in relation to long‐term cardiovascular health. Our findings highlight the need for further research that investigates potential mechanisms of these associations so that prevention and intervention strategies can target key factors to reduce the impact of childhood trauma, particularly in LMICs where trauma exposure is highly prevalent. Research has already provided some evidence of the success of these strategies in Pelotas. The Pelotas Pact for Peace (https://peaceinourcities.org/cities/pelotas‐brazil/) is a city‐led intervention programme launched in 2017 to reduce urban crime and violence through projects spanning health, education and the criminal justice system. Reductions in violent crime have been observed following the Pact (Degli Esposti et al., 2023), highlighting the strengths of a coordinated and interdisciplinary strategy to mitigate trauma exposure in LMICs. Future research should investigate the broader health‐related benefits of such a coordinated approach to examine whether outcomes for young people improve in the longer term.

## Ethical considerations

Assessments at all follow‐ups were approved by research ethics boards at the Federal University of Pelotas and the University of São Paulo, and cohort participants/caregivers provided written informed assent/consent as appropriate.

**Key points**

- Childhood trauma has been linked with an increased risk of substance use and subsequent poor cardiovascular health. However, longitudinal evidence in LMICs is scarce.
- In this Brazilian birth cohort (*N* = 4,229), more than 80% of adolescents were trauma‐exposed by age 18, with 25.3% of adolescents reporting exposure to three or more traumas during their lifetime.
- Greater cumulative trauma exposure was both concurrently and longitudinally associated with increased odds of alcohol, smoking and illicit drug use at age 18.
- Unexpectedly, greater trauma exposure was associated with lower resting HR and BP. Substance use partially mediated these associations.
- Prevention and intervention strategies targeting childhood trauma are critical given its high prevalence and robust links to substance use behaviours; further exploration of associations with cardiovascular health indicators is warranted.

## Supporting information


**Appendix S1.** Supplementary methods.

**Appendix S2.** Supplementary analyses.

**Appendix S3.** Brazilian Portuguese abstract translation.

**Appendix S4.** STROBE statement—checklist of items that should be included in reports of observational studies.

## Data Availability

Applications to use the data can be made by contacting the researchers of the 2004 Pelotas Birth Cohort (https://epidemio‐ufpel.org.br/corpo‐docente‐ppgep/). A list of administered questionnaires at each timepoint and relevant application forms can be accessed online (https://epidemio‐ufpel.org.br/coorte‐2004/). Researchers with successful applications will receive a dataset including the requested variables and unique participant IDs. The analysis code used in this study is available through a public GitHub repository at https://github.com/megan‐l‐bailey/2004Pelotas‐Trauma‐HRB‐CVI.
