# Supplementary material for: Childhood trauma, adolescent risk behaviours and cardiovascular health indices in the 2004 Pelotas Birth Cohort
Source: J Child Psychol Psychiatry. 2025 Apr 30;66(11):1653–63. doi: 10.1111/jcpp.14173 (PMC12571934; doi:10.1111/jcpp.14173)
Supplement: Supplementary file 1 — Appendix S1. Supplementary methods. [file JCPP-66-1653-s001.docx]

**Childhood trauma, adolescent risk behaviours, and cardiovascular health indices in the 2004 Pelotas Birth Cohort**

**Supplement 1 – Methods**

**Supplementary Methods**

**Study Setting**

Pelotas is the fourth most populous city in Brazil’s southernmost state, Rio Grande do Sul, with a population of ~325,000 (<https://cidades.ibge.gov.br/brasil/rs/pelotas/panorama>; accessed 02/09/2024). Approximately 93% of the population live in the urban city area. In 2010, the Pelotas Gini index was 56·0, indicating significant income inequality, and higher than the Brazilian national average of 48·9 in the same year (<http://tabnet.datasus.gov.br/cgi/ibge/censo/cnv/ginibr.def>; accessed 02/09/2024). Furthermore, 2010 census data indicated that the Human Development Index (HDI) in Pelotas was high (0·739) and was comparable to the state (0·771) and Brazil as a whole (0·726). In 2021, GDP per capita for Pelotas was 5551·39 USD, lower than the national GDP per capita of 7481·68 USD (<https://www.ibge.gov.br/en/cities-and-states/rs/pelotas.html>; accessed 02/09/2024).

**Cohort Details**

Of 4263 live births, 4231 infants were recruited into the sample at birth (99·2%). Ninety-five deaths were recorded prior to the 6-year follow-up. The 6-year follow-up took place from 11^th^ October 2010 to 8^th^ August 2011. Of 4136 eligible children, 3722 participated in the follow-up (90·0%), and 414 refused or were lost to follow-up (10·0%). Children were aged, on average, 6·70 years (SD=0·20). Three deaths were recorded between the 6- and 11-year follow-ups. The 11-year follow-up took place from 3^rd^ February 2015 to 25^th^ September 2015. Of 4133 eligible children, 3566 participated in the follow-up (86·3%), and 567 refused or were lost to follow-up (13·7%). Children were aged, on average, 10·88 years (SD=0.26). Four deaths were recorded between the 11- and 15-year follow-ups. The 15-year follow-up took place from 20^th^ November 2019 to 17^th^ March 2020 (data collection was suspended early due to the COVID-19 pandemic). Of 4129 eligible children, 1949 participated in the follow-up (47·2%), and 2180 refused or were lost to follow-up (52·8%). Children were aged, on average, 15·69 years (SD=0·20). Five deaths were recorded between the 15- and 18-year follow-ups. The 18-year follow-up took place from 21^st^ February 2022 to 30^th^ December 2022. Of 4124 eligible children, 3489 participated in the follow-up (84·6%), and 635 refused or were lost to follow-up (15·4%). Children were aged, on average, 17·97 years (SD=0·29).

**Trauma Exposure Questionnaire Items at Ages 15 and 18**

At age 15, nine survey items were used to assess adolescent reports of lifetime trauma exposure. Three items (exposure to a robbery, serious accident, and parental death) were assessed via a general questionnaire administered to the adolescent by an interviewer; and six items were assessed using a confidential questionnaire completed independently by the adolescent (deliberate attack, attacked without a weapon, attacked by a group/gang, physical abuse, sexual abuse, and witnessed domestic violence). At age 18, lifetime trauma exposure was captured using the adolescent’s response to a single binary question on the Mini-International Neuropsychiatric Interview (MINI; ‘Have you ever experienced or witnessed or had to deal with an extremely traumatic event that included actual or threatened death or serious injury or sexual violence to you or someone else?’; Sheehan et al., 1998) as well as six additional questionnaire items. Three items were assessed via a general questionnaire administered to the adolescent by an interviewer (attack/threat, serious accident, and parental death); and three items were assessed via a confidential questionnaire completed independently by adolescents (physical abuse, sexual abuse, and witnessed domestic violence).

**Cumulative Trauma – Likelihood-Ratio Tests**

The total number of trauma exposures was recoded into four categories (0 exposures, 1 exposure, 2 exposures, and ≥3 exposures) due to low frequencies at the upper extreme. We conducted likelihood-ratio tests (using complete case data) to determine whether the association between cumulative trauma and the log-odds of each outcome (problematic alcohol use, smoking, illicit drug use, sleep duration, resting heart rate (HR), systolic blood pressure (BP), and diastolic BP) was linear (i.e. by comparing the likelihood in a model that treated cumulative trauma as a numeric exposure and a model that treated cumulative trauma as a categorical exposure). All tests were non-significant, providing no evidence against the null hypothesis that the relationship between cumulative trauma and the log-odds of the outcome were linear (*p*-values ranged from 0·233 to 0·982). We therefore treated cumulative trauma as a numeric exposure variable (with regression coefficients representing a one-category increase in cumulative trauma).

**Sleep Duration – Assessment Details**

Data for average nightly sleep duration up to age 18 were manually inspected for potential errors (e.g., 24-hour time errors) and corrected as necessary. Individuals reporting <3 hours or >13 hours were excluded. We also used plots to examine whether there was a linear relationship between cumulative trauma and sleep duration, to ensure the assumptions of linear regression were met (linearity and homoskedasticity). There was no indication that these assumptions were not met, and we therefore assumed a linear relationship.

**Confounders – Assessment Details**

Adolescent sex was coded as male or female. Adolescent ethnicity was coded as White or other. Maternal smoking during pregnancy was coded as present or absent and defined as at least one cigarette daily during any trimester of pregnancy. Maternal alcohol consumption during pregnancy was coded as present or absent and defined as any alcohol intake during any trimester of pregnancy. Maternal education at birth was coded continuously as number of school years completed. Monthly family income (in Brazilian Reals, R$) at birth was treated as a continuous variable. The day of the year the adolescent was born was used as a proxy for cohort birth order. Adolescents born on January 1^st^, 2004, were coded as 0, through to those born on December 31^st^, 2004, who were coded as 365. This was because each follow-up was conducted in birth order, with cohort members born in January contacted first; after a first attempt at contact was made, each individual was contacted a further three times to participate in the follow-up before a visit to their home address was arranged. Due to the COVID-19 pandemic, research procedures for the 15-year follow-up were permanently suspended, at which point all adolescents had been contacted once, and researchers were in the process of recontacting individuals for the first time. As such, birth order had some influence on the likelihood of having missing data at age 15 (in addition to analysis model outcomes), and we therefore adjusted for this in all analyses to avoid bias. Physical activity was measured via adolescent self-reports of time spent playing 13 different sports in the last week; data were manually inspected for potential errors (e.g., hour and minute errors) and corrected as necessary.

Confounders were chosen *a priori* based on a directed acyclic graph (DAG) rather than using a data driven approach. However, associations between the confounders and our main exposure and outcomes at age 18 are presented in Table S1 (p 8).

**Open Science Framework Pre-registration**

This study was pre-registered on the Open Science Framework (<https://doi.org/10.17605/OSF.IO/BG4EH>). We made some changes to our analysis plans. First, we did not investigate the proportion of insufficient sleep duration that was attributable to childhood trauma up to age 18 by calculating the population attributable fraction. This would have required the dichotomisation of the sleep duration variable (<7 hours of sleep=insufficient sleep). However, recent research indicates that childhood trauma may also be related to long sleep duration (Schneiderman, Ji, Susman, & Negriff, 2018). We therefore felt that dichotomising sleep duration could preclude investigating the nuances of the relationship between childhood trauma and sleep duration and decided not to conduct this analysis, especially as the population attributable fraction estimate would then be based on a different analysis compared to our main analysis. Second, we did not include sleep duration as a mediator in our final model. In our regression analyses, we found that sleep duration was unrelated to both cumulative trauma and the cardiovascular health indices and felt that the final model would conceptually make more sense if it focused just on the three substance use mediators (problematic alcohol use, smoking, and illicit drug use).

**Missing Data**

Missingness for cumulative trauma was 15·8% at age 11, 54·6% at age 15, and 25·2% at age 18. Missingness for risk behaviours was 24·9% for problematic alcohol use, 20·6% for current smoking, 22·6% for current illicit drug use, and 27·4% for sleep duration at age 18. A summary of missing data for all analysis variables, covariates, and auxiliary variables is presented in Table S2 (p 9-10).

A comparison of complete cases and those with missing data for trauma exposure and/or risk behaviours at ages 15 and 18 is presented in Tables S3 and S4 (p 11-12). In general, complete case analyses are valid if the analysis model outcome is unrelated to missingness, conditional on the analysis model covariates (Hughes, Heron, Sterne, & Tilling, 2019). Although we cannot examine the association between the outcome and missingness using observed data, we can examine whether this assumption is violated due to a covariate that is associated with both missingness and the analysis model outcome (problematic alcohol use, smoking, illicit drug use, and sleep duration at age 18). Maternal smoking during pregnancy and maternal education were both associated with at least one outcome variable and with missingness. Given that these variables were also considered confounders for the trauma-risk behaviour associations, we adjusted for these variables to avoid bias from both selective attrition and confounding. Cohort birth order was also associated with at least one outcome variable and missingness; we therefore adjusted for this. Finally, lifetime alcohol use and smoking at age 11 were both associated with at least one outcome variable and missingness. However, we were unable to condition on these variables as they are likely to be on the causal pathway between childhood trauma and risk behaviours at age 18. Complete case analyses may therefore be biased, but these variables were used as auxiliary variables in our imputation models to reduce this bias.

We used multivariate imputation by chained equations with 50 imputed datasets to address missing data (N=4229; van Buuren & Oudshoorn, 2000). Auxiliary variables included: alcohol use at ages 11 (lifetime use) and 15 (ever been drunk), lifetime smoking at ages 11 and 15, lifetime illicit drug use at age 15, sleep duration (hours) at ages 11 and 15, physical activity (number of activities) at age 11, age-adjusted BMI at age 11, birthweight (grams), and total score on the Conflict Tactics Scale Parent-Child Version (CTSPC; Straus, Hamby, Finkelhor, Moore, & Runyan, 1998) at ages 6 and 11. As trauma variables reflected lifetime trauma exposures up to each age, rather than traumas reported at each age, we encountered problems with perfect prediction when imputing all trauma variables in the same model. As trauma exposure up to different ages were never used together within the same analysis model, we were able to solve this issue by performing 4 different imputation models for our main analyses. One model imputed binary trauma exposure up to age 18 (for sample characteristics and PAF estimation). Three models imputed cumulative trauma up to ages 11, 15, and 18 years with all necessary variables. A fifth imputation model was derived for our mediation analyses, including cumulative trauma up to age 15, risk behaviours at age 18, and resting HR, systolic BP, and diastolic BP at age 18. Only data for a subsample of cohort members with complete resting HR data at age 18 (*n*=3196) was imputed in this model given insufficient auxiliary variables to impute resting HR. All binary variables were imputed using logistic regression and all continuous variables were imputed using predictive mean matching. Variables were imputed by sex, enabling the examination of sex differences. Stata do-files, detailing imputation model inclusions and omissions, can be provided on request by contacting the corresponding author (MB). All imputation models were examined for convergence. All Monte Carlo errors for effect estimates were less than 10% of its standard error, suggesting 50 imputed datasets was sufficient (White, Royston, & Wood, 2011).

**References**

Hughes, R. A., Heron, J., Sterne, J. A. C., & Tilling, K. (2019). Accounting for missing data in statistical analyses: multiple imputation is not always the answer. *International Journal of Epidemiology, 48*(4), 1294-1304.

Schneiderman, J. U., Ji, J., Susman, E. J., & Negriff, S. (2018). Longitudinal Relationship Between Mental Health Symptoms and Sleep Disturbances and Duration in Maltreated and Comparison Adolescents. *Journal of Adolescent Health, 63*(1), 74-80.

Sheehan, D. V., Lecrubier, Y., Sheehan, K. H., Amorim, P., Janavs, J., Weiller, E., et al. (1998). The Mini-International Neuropsychiatric Interview (M.I.N.I.): the development and validation of a structured diagnostic psychiatric interview for DSM-IV and ICD-10. *Journal of Clinical Psychiatry, 59*(20), 22-33.

Straus, M. A., Hamby, S. L., Finkelhor, D., Moore, D. W., & Runyan, D. (1998). Identification of Child Maltreatment With the Parent-Child Conflict Tactics Scales: Development and Psychometric Data for a National Sample of American Parents. *Child Abuse & Neglect, 22*(4), 249-270.

van Buuren, S., & Oudshoorn, C. G. M. (2000). *Multivariate imputation by chained equations*. Leiden: TNO Institute.

White, I. R., Royston, P., & Wood, A. M. (2011). Multiple imputation using chained equations: Issues and guidance for practice. *Statistics in Medicine, 30*(4), 377-399.

**Table S1.** Associations between confounders and the main exposure and outcomes at age 18

|  | **Cumulative trauma** | | **Problematic alcohol use** | | **Smoking** | | **Illicit drug use** | | **Sleep duration** | |
| --- | --- | --- | --- | --- | --- | --- | --- | --- | --- | --- |
|  | B (95% CI) | *p* | OR (95% CI) | *p* | OR (95% CI) | *p* | OR (95% CI) | *p* | B (95% CI) | *p* |
| **Confounders** | | | | | | | | | | |
| Male sex | 0·05 (-0·04, 0·13) | 0·284 | 1·15 (0·97, 1·35) | 0·114 | 1·07 (0·80, 1·42) | 0·663 | 1·17 (0·98, 1·41) | 0·081 | -0·41 (-0·51, -0·30) | <0·001 |
| Adolescent ethnicity (White) | -0·11 (-0·20, -0·02) | 0·015 | 0·80 (0·67, 0·95) | 0·012 | 0·88 (0·65, 1·20) | 0·434 | 0·79 (0·65, 0·95) | 0·014 | 0·04 (-0·08, 0·15) | 0·540 |
| Maternal smoking during pregnancy (yes) | 0·31 (0·21, 0·40) | <0·001 | 1·52 (1·26, 1·83) | <0·001 | 2·52 (1·88, 3·38) | <0·001 | 1·28 (1·05, 1·57) | 0·014 | 0·003 (-0·12, 0·12) | 0·958 |
| Maternal alcohol consumption during pregnancy (yes) | 0·49 (0·26, 0·72) | <0·001 | 3·00 (1·94, 4·63) | <0·001 | 2·00 (1·06, 3·74) | 0·031 | 1·53 (0·96, 2·42) | 0·072 | 0·06 (-0·23, 0·36) | 0·680 |
| Monthly family income | -0·00003 (-0·0001, 0.00001) | 0·130 | 1·00 (1·00, 1·00) | 0·959 | 1·00 (1·00, 1·00) | 0·001 | 1·00 (1·00, 1·00) | 0·014 | -0·00003 (-0·0001, 0·00002) | 0·219 |
| Maternal education | -0·02 (-0·04, -0·01) | <0·001 | 0·97 (0·95, 1·00) | 0·042 | 0·87 (0·83, 0·91) | <0·001 | 0·99 (0·96, 1·01) | 0·316 | -0·02 (-0·04, -0·01) | 0·007 |
| Cohort birth order^a^ | -0·002 (-0·002, -0·001) | <0·001 | 1·00 (1·00, 1·00) | 0·098 | 1·00 (1·00, 1·00) | 0·068 | 1·00 (1·00, 1·00) | 0·118 | -0·001 (-0·001, -0·0003) | 0·001 |
| **Auxiliary Variables** | | | | | | | | | | |
| Lifetime alcohol use at age 11 | 0·36 (0·19, 0·52) | <0·001 | 1·86 (1·36, 2·53) | <0·001 | 1·52 (0·92, 2·50) | 0·105 | 1·12 (0·79, 1·60) | 0·511 | -0·13 (-0·34, 0·08) | 0·215 |
| Lifetime smoking at age 11 | 0·69 (0·28, 1·09) | 0·001 | 1·58 (0·73, 3·42) | 0·245 | 3·59 (1·43, 9·02) | 0·006 | 2·20 (1·02, 4·78) | 0·045 | -0·10 (-0·62, 0·42) | 0·716 |
| Sleep duration at age 11 | -0·03 (-0·05, -0·001) | 0·039 | 0·96 (0·91, 1·01) | 0·096 | 0·98 (0·90, 1·07) | 0·657 | 1·01 (0·95, 1·07) | 0·773 | 0·06 (0·03, 0·09) | <0·001 |

*Note*. Based on complete case data for trauma and risk behaviours at age 18 and baseline confounders (*n*=2574). Coefficients for cumulative trauma and sleep duration are unstandardised beta and coefficients for substance use behaviours are odds ratios. *Key*: CI=confidence interval.

**Table S2.** Summary of missing data

| **Variable** | **Description** | **Missing values** | **Range** | **Mean** | **SD** | **Skewness** | **Kurtosis** |
| --- | --- | --- | --- | --- | --- | --- | --- |
| **Main Analysis Variables** | | | | | |  |  |
| y18_alcohol | Problematic alcohol use at age 18 | 1054 (24·9%) | 0/1 |  |  |  |  |
| y18_smoking | Current smoking status at age 18 | 872 (20·6%) | 0/1 |  |  |  |  |
| y18_druguse | Current illicit drug use at age 18 | 957 (22·6%) | 0/1 |  |  |  |  |
| y18_sleepdur | Average sleep duration at age 18 | 1158 (27·4%) | 3·12-12·92 | 7·42 | 1·41 | <0·001 | 0·045 |
| y11_cte | Cumulative trauma up to age 11 | 667 (15·8%) | 0-3 | 0·52 | 0·84 | <0·001 | <0·001 |
| y15_cte | Cumulative trauma up to age 15 | 2311 (54·6%) | 0-3 | 1·52 | 1·11 | 0·379 | <0·001 |
| y18_cte | Cumulative trauma up to age 18 | 1067 (25·2%) | 0-3 | 1·51 | 1·06 | 0·004 | <0·001 |
| y18_alltrauma | Any trauma exposure up to age 18 | 1067 (25·2%) | 0/1 |  |  |  |  |
| **Mediation Analysis Variables** | | | | | | | |
| y18_hr | Resting HR at age 18 | 1033 (24·4%) | 45-183·5 | 80·43 | 14·51 | <0·001 | <0·001 |
| y18_sbp | Resting systolic BP at age 18 | 1033 (24·4%) | 82·5-179 | 122·88 | 13·64 | <0·001 | 0·001 |
| y18_dbp | Resting diastolic BP at age 18 | 1033 (24·4%) | 44·5-109 | 70·10 | 8·38 | <0·001 | <0·001 |
| y15_activity | Physical activity at age 15 (hours) | 2846 (67·3%) | 0·05-94 | 8·56 | 9·98 | <0·001 | <0·001 |
| y15_bmi | BMI at age 15 (age-adjusted z-score) | 2326 (55·0%) | -3·69-4·87 | 0·45 | 1·34 | 0·005 | 0·002 |
| **Confounders** | | | | | |  |  |
| sex | Sex of child | 0 | 0/1 |  |  |  |  |
| ethnicity | Adolescent ethnicity | 232 (5·5%) | 0/1 |  |  |  |  |
| msmoking | Maternal smoking in any trimester | 0 | 0/1 |  |  |  |  |
| malcohol | Maternal alcohol consumption during pregnancy | 0 | 0/1 |  |  |  |  |
| meduc | Maternal education | 43 (1·0%) | 0-18 | 8·09 | 3·47 | 0·021 | <0·001 |
| fincome | Total family income at birth | 0 | 0-22000 | 803·59 | 1109·06 | <0·001 | <0·001 |
| birthday | Ranked date of birth | 0 | 0-365 | 179·88 | 103·95 | 0·585 | <0·001 |
| **Auxiliary Variables** | | | | | |  |  |
| y11_alcohol | Lifetime alcohol use at age 11 | 744 (17·6%) | 0/1 |  |  |  |  |
| y11_smoking | Lifetime smoking status at age 11 | 738 (17·5%) | 0/1 |  |  |  |  |
| y11_sleepdur | Average sleep duration at age 11 | 966 (22·8%) | 3-16·5 | 9·29 | 1·77 | 0·643 | 0·004 |
| y15_alcohol | Lifetime drunk at age 15 | 2363 (55·9%) | 0/1 |  |  |  |  |
| y15_smoking | Lifetime smoking at age 15 | 2328 (55·0%) | 0/1 |  |  |  |  |
| y15_druguse | Lifetime illicit drug use at age 15 | 2366 (55·9%) | 0/1 |  |  |  |  |
| y15_sleepdur | Average sleep duration at age 15 | 2418 (57·2%) | 3·19-13·10 | 7·55 | 1·49 | <0·001 | 0·006 |
| y11_activity | Tally of physical activities at age 11 | 698 (16·5%) | 0-10 | 0·42 | 0·87 | <0·001 | <0·001 |
| y11_bmi | BMI at age 11 (age-adjusted z-score) | 762 (18·0%) | -3·87-5·48 | 0·74 | 1·41 | 0·018 | <0·001 |
| birthweight | Birth weight (grams) | 3 (0·1%) | 450-5995 | 3150·13 | 566·19 | <0·001 | <0·001 |
| tctspc6^a^ | CTSPC total score at age 6 years | 769 (18·2%) | 0-12 | 3·73 | 2·28 | <0·001 | 0·380 |
| tctspc11^a^ | CTSPC total score at age 11 years | 705 (16·7%) | 0-14 | 3·64 | 2·43 | <0·001 | 0·027 |

^a^CTSPC=Conflict Tactics Scale: Parent-Child Version; total score sums items across the psychological aggression, corporal punishment, and physical maltreatment subscales (exclusion of non-violent discipline subscale). *Key:* BP=blood pressure. HR=heart rate.

**Table S3.** Comparison of complete cases and those with missing information for trauma exposure at age 15 and adolescent risk behaviours at age 18

| **Variable** | **Proportion missingness** | **Complete Cases (*n*=1477)** | | **Missing Cases (*n*=2752)** | | **Association with missingness** | |
| --- | --- | --- | --- | --- | --- | --- | --- |
|  | *n*(%) | *n* | (%) or M(SD) | *n* | (%) or M(SD) | OR (95% CI) | *p*-value |
| **Exposure** |  |  |  |  |  |  |  |
| Trauma exposed at age 6 | 646 (15·3) | 178 | 12·7 | 273 | 12·5 | 0·98 (0·80-1·20) | 0·844 |
| Trauma exposed at age 11 | 667 (15·8) | 482 | 33·8 | 735 | 34·4 | 1·03 (0·90-1·19) | 0·671 |
| Lifetime alcohol use at age 11 | 744 (17·6) | 115 | 8·2 | 164 | 7·9 | 0·97 (0·75-1·24) | 0·788 |
| Lifetime smoking at age 11 | 738 (17·5) | 18 | 1·3 | 31 | 1·5 | 1·18 (0·65-2·11) | 0·589 |
| Sleep duration at age 11 | 966 (22·8) | 1345 | 9·25 (1·75) | 1918 | 9·32 (1·79) | 1·02 (0·98-1·06) | 0·259 |
| **Confounders** |  |  |  |  |  |  |  |
| Male sex | 0 (0) | 758 | 51·3 | 1436 | 52·2 | 1·04 (0·91-1·17) | 0·594 |
| Adolescent ethnicity (white) | 232 (5·5) | 1005 | 68·5 | 1720 | 68·0 | 0·98 (0·85-1·12) | 0·769 |
| Maternal smoking during pregnancy | 0 (0) | 376 | 25·5 | 786 | 28·6 | 1·17 (1·01-1·35) | 0·031 |
| Maternal alcohol use during pregnancy | 0 (0) | 51 | 3·5 | 89 | 3·2 | 0·93 (0·66-1·33) | 0·704 |
| Maternal education (years at birth) | 43 (1·0) | 1466 | 8·36 (3·37) | 2720 | 7·95 (3·52) | 0·97 (0·95-0·98) | <0·001 |
| Monthly family income (BRL at birth) | 0 (0) | 1477 | 838·06 (1135·20) | 2752 | 785·08 (1094·53) | 1·00 (1·00-1·00) | 0·141 |
| Cohort birth order^a^ | 0 (0) | 1477 | 142·26 (89·79) | 2752 | 200·08 (105·42) | 1·01 (1·00-1·01) | <0·001 |

*Note*. ^a^Ranked date of birth relative to other cohort members (lower numbers correspond to birth earlier in the year). *Key:* BRL=Brazilian Real. CI=confidence interval. M=mean. OR=odds ratio. SD=standard deviation.

**Table S4.** Comparison of complete cases and those with missing information for trauma exposure and/or adolescent risk behaviours at age 18

| **Variable** | **Proportion missingness** | **Complete Cases (*n*=2635)** | | **Missing Cases (*n*=1594)** | | **Association with missingness** | |
| --- | --- | --- | --- | --- | --- | --- | --- |
|  | *n*(%) | *n* | (%) or M(SD) | *n* | (%) or M(SD) | OR (95% CI) | *p*-value |
| **Exposure** |  |  |  |  |  |  |  |
| Trauma exposed at age 6 | 646 (15·3) | 308 | 12·6 | 143 | 12·5 | 0·99 (0·80-1·22) | 0·925 |
| Trauma exposed at age 11 | 667 (15·8) | 826 | 33·5 | 391 | 35·7 | 1·10 (0·95-1·28) | 0·196 |
| Lifetime alcohol use at age 11 | 744 (17·6) | 176 | 7·2 | 103 | 9·9 | 1·41 (1·09-1·81) | 0·009 |
| Lifetime smoking at age 11 | 738 (17·5) | 27 | 1·1 | 22 | 2·1 | 1·93 (1·10-3·41) | 0·023 |
| Sleep duration at age 11 | 966 (22·8) | 2307 | 9·26 (1·74) | 956 | 9·36 (1·86) | 1·03 (0·99-1·08) | 0·136 |
| **Confounders** |  |  |  |  |  |  |  |
| Male sex | 0 (0) | 1350 | 51·2 | 844 | 53·0 | 1·07 (0·95-1·21) | 0·279 |
| Adolescent ethnicity (white) | 232 (5·5) | 1796 | 69·1 | 929 | 66·6 | 0·89 (0·78-1·02) | 0·105 |
| Maternal smoking during pregnancy | 0 (0) | 686 | 26·0 | 476 | 29·9 | 1·21 (1·05-1·39) | 0·007 |
| Maternal alcohol use during pregnancy | 0 (0) | 88 | 3·3 | 52 | 3·3 | 0·98 (0·69-1·38) | 0·892 |
| Maternal education (years at birth) | 43 (1·0) | 2607 | 8·39 (3·34) | 1579 | 7·60 (3·63) | 0·94 (0·92-0·95) | <0·001 |
| Monthly family income (BRL at birth) | 0 (0) | 2635 | 831·42 (1091·11) | 1594 | 757.57 (1136·96) | 1·00 (1·00-1·00) | 0·037 |
| Cohort birth order^a^ | 0 (0) | 2635 | 184·88 (103·53) | 1594 | 171·62 (104·15) | 1·00 (1·00-1·00) | <0·001 |

*Note*. ^a^Ranked date of birth relative to other cohort members (lower numbers correspond to birth earlier in the year). *Key:* BRL=Brazilian Real. CI=confidence interval. M=mean. OR=odds ratio. SD=standard deviation.
