# Supplementary material for: Childhood trauma, adolescent risk behaviours and cardiovascular health indices in the 2004 Pelotas Birth Cohort
Source: J Child Psychol Psychiatry. 2025 Apr 30;66(11):1653–63. doi: 10.1111/jcpp.14173 (PMC12571934; doi:10.1111/jcpp.14173)
Supplement: Supplementary file 2 — Appendix S2. Supplementary analyses. [file JCPP-66-1653-s004.docx]

**Childhood trauma, adolescent risk behaviours, and cardiovascular health indices in the 2004 Pelotas Birth Cohort**

**Supplement 2 – Analyses**

**Supplementary Imputed Analyses**

**Sex Differences**

Three interaction effects were found to be significant. The cross-sectional association between cumulative trauma up to age 18 and current illicit drug use at age 18 significantly differed by sex (interaction effect *p*-value=0·041). Sex-stratified analyses revealed that the OR for cumulative trauma in males (adjusted OR=1·36 [95% CI 1·22-1·52], *p<*0·001) was greater than the OR in females (1·19 [1·07-1·32], *p*=0·002). Similarly, the longitudinal association between cumulative trauma up to age 11 and current illicit drug use at age 18 significantly differed by sex (interaction effect *p*-value=0·012). Sex-stratified analyses revealed that the OR for males (1·26 [1·11-1·42], *p*<0·001) was greater than the OR for females (1·00 [0·88-1·15], *p*=0·957). Finally, the longitudinal association between cumulative trauma up to age 15 and current smoking at age 18 significantly differed by sex (interaction effect *p*-value=0·035). Sex-stratified analyses revealed that the OR for males (1·71 [1·36-2·16], *p*<0·001) was greater than the OR for females (1·21 [0·98-1·49], *p*=0·074). Thus, where interaction effects were observed, these suggested that cumulative trauma increased the odds of adolescent risk behaviours in males more than females.

**Sensitivity Mediation Analyses**

Table S3 (p 6) shows the total causal effect, pure natural direct effect, and total natural indirect effect of cumulative trauma up to age 15 on resting heart rate (HR) via each mediator examined individually in three single mediator models. There was some evidence of inconsistent mediation: indirect effect coefficients for problematic alcohol use and illicit drug use were both negative, while the coefficient for smoking was positive. In adjusted models, there was little evidence of an indirect effect via smoking or illicit drug use at age 18 individually. However, there was some evidence of a small natural indirect effect via problematic alcohol use (*B*=-0·09 [95% CI -0·17, -0·02]), comprising 7·6% of the total effect.

Table S4 (p 7) shows the total causal effect, pure natural direct effect, and total natural indirect effect of cumulative trauma up to age 18 on resting systolic blood pressure (BP) via each mediator individually. There was no inconsistent mediation. In adjusted models, there was little evidence of an indirect effect via problematic alcohol use or illicit drug use at age 18 individually. However, there was some evidence of a small natural indirect effect via smoking (*B*=-0·10 [95% CI -0·17, -0·03]), comprising 16·4% of the total effect.

Table S5 (p 8) shows the total causal effect, pure natural direct effect, and total natural indirect effect of cumulative trauma up to age 18 on resting diastolic BP via each mediator individually. There was no inconsistent mediation. In adjusted models, there was little evidence of an indirect effect via illicit drug use at age 18. However, there was some evidence of a small natural indirect effect via both problematic alcohol use (*B*=-0·04 [95% CI -0·08, -0·01]) and smoking (*B*=-0·05 [-0·09, -0·02]) individually, comprising 6·2% and 7·5% of the total effects respectively.

**Table S1.** Cross-sectional associations between trauma exposure up to age 18, coded as a binary variable, and adolescent risk behaviours at age 18

|  | **Total Sample** | **Trauma up to age 18** | | **Coefficient (95% CI)** | ***p*** |
| --- | --- | --- | --- | --- | --- |
|  |  | **Unexposed (18·9%)** | **Exposed (81·1%)** |  |  |
| **Binary risk behaviours** | | | | | |
| Problematic alcohol use^a^ | 30·3 (0·01) | 18·5 (0·02) | 33·0 (0·01) | 2·06 (1·65, 2·57) | <0·001 |
| Current smoking | 8·6 (0·005) | 3·3 (0·01) | 9·8 (0·01) | 2·98 (1·77, 5·02) | <0·001 |
| Current illicit drug use | 27·1 (0·01) | 18·8 (0·02) | 29·1 (0·01) | 1·66 (1·31, 2·11) | <0·001 |
| **Continuous risk behaviours** | | | | | |
| Sleep duration (hours) | 7·45 (0·02) | 7·47 (0·06) | 7·42 (0·03) | -0·06 (-0·19, 0·07) | 0·389 |

*Note.* Based on imputed data (*N*=4229). Data for binary risk behaviours are % (SE); coefficients are odds ratios. Data for continuous risk behaviours are mean (SE); coefficients are unstandardised beta. Analyses were adjusted for baseline confounders (child sex, child ethnicity, maternal smoking during pregnancy, maternal alcohol consumption during pregnancy, maternal education at birth, monthly family income at birth, and cohort birth order). ^a^Based on the Alcohol Use Disorders Identification Test [(AUDIT);](https://assets.publishing.service.gov.uk/media/6357a7af8fa8f557d85b7c44/Alcohol-use-disorders-identification-test-AUDIT_for-print.pdf) adolescents scoring 8 and above were coded as having problematic alcohol use. *Key*: CI=confidence interval.

**Table S2.** Descriptive statistics for adolescent risk behaviours and cardiovascular health indices according to cumulative trauma exposure up to age 15

|  | **Total Sample** | **Cumulative trauma exposure up to age 15** | | | |
| --- | --- | --- | --- | --- | --- |
|  |  | **0 traumas (22·1%)** | **1 trauma (31·2%)** | **2 traumas (21·0%)** | **≥3 traumas (25·7%)** |
| **Binary variables** | | | | | |
| Problematic alcohol use at age 18^a^ | 30·1 (0·01) | 21·7 (0·02) | 28·2 (0·02) | 32·6 (0·02) | 38·2 (0·02) |
| Current smoking at age 18 | 8·1 (0·005) | 4·7 (0·01) | 6·4 (0·01) | 8·2 (0·01) | 13·0 (0·01) |
| Current illicit drug use at age 18 | 26·8 (0·01) | 21·3 (0·02) | 23·7 (0·02) | 28·4 (0·02) | 34·0 (0·02) |
| **Continuous variables** | | | | | |
| Sleep duration at age 18 (hours) | 7·40 (0·03) | 7·49 (0·07) | 7·38 (0·06) | 7·38 (0·06) | 7·35 (0·06) |
| Resting HR at age 18 (bpm) | 80·43 (0·26) | 82·95 (0·71) | 80·63 (0·57) | 79·62 (0·67) | 78·69 (0·60) |
| Resting systolic BP at age 18 (mmHg) | 122·88 (0·24) | 123·08 (0·62) | 123·52 (0·53) | 123·16 (0·65) | 121·71 (0·60) |
| Resting diastolic BP at age 18 (mmHg) | 70·10 (0·15) | 71·18 (0·38) | 70·44 (0·33) | 69·78 (0·38) | 69·03 (0·34) |
| Physical activity at age 15 (hours) | 8·37 (0·25) | 7·71 (0·49) | 8·62 (0·42) | 8·44 (0·49) | 8·58 (0·51) |
| BMI at age 15 (age-adjusted z-score) | 0·49 (0·03) | 0·48 (0·06) | 0·47 (0·05) | 0·52 (0·06) | 0·50 (0·06) |

*Note.* Based on imputed data (*n*=3196). Data are % (SE) for binary variables and mean (SE) for continuous variables. ^a^Based on the Alcohol Use Disorders Identification Test [(AUDIT);](https://assets.publishing.service.gov.uk/media/6357a7af8fa8f557d85b7c44/Alcohol-use-disorders-identification-test-AUDIT_for-print.pdf) adolescents scoring ≥8 were coded as having problematic alcohol use. *Key*: BP=diastolic blood pressure. HR=heart rate.

**Table S3.**  Mediation models of the effect of cumulative trauma up to age 15 on resting heart rate at age 18 through mediating substance use behaviours (problematic alcohol use, smoking, and illicit drug use; included individually)

|  | **Unadjusted** | **Adjusted** |
| --- | --- | --- |
| **Problematic alcohol use** | | |
| Total causal effect | -1·35 (-2·01, -0·68) | -1·23 (-1·84, -0·63) |
| Natural direct effect | -1·19 (-1·85, -0·54) | -1·14 (-1·74, -0·54) |
| Natural indirect effect | -0·15 (-0·24, -0·06) | -0·09 (-0·17, -0·02) |
| Proportion mediated | 11·4% | 7·6% |
| **Smoking** | | |
| Total causal effect | -1·34 (-2·00, -0·67) | -1·23 (-1·84, -0·63) |
| Natural direct effect | -1·33 (-2·00, -0·66) | -1·24 (-1·84, -0·64) |
| Natural indirect effect | -0·003 (-0·07, 0·07) | 0·01 (-0·06, 0·05) |
| Proportion mediated | 0·2% | -0·01% |
| **Illicit drug use** | | |
| Total causal effect | -1·34 (-2·01, -0·68) | -1·23 (-1·84, -0·63) |
| Natural direct effect | -1·29 (-1·96, -0·61) | -1·22 (-1·82, -0·61) |
| Natural indirect effect | -0·05 (-0·12, 0·01) | -0·02 (-0·07, 0·03) |
| Proportion mediated | 4·0% | 1·3% |

*Note*. Based on imputed data (*n*=3196). Baseline confounders include child sex, child ethnicity, maternal smoking during pregnancy, maternal alcohol consumption during pregnancy, maternal education at birth, monthly family income at birth, and cohort birth order (lower numbers correspond to birth earlier in the year). Intermediate confounders include age-adjusted BMI and estimated hours of physical activity at age 15.

**Table S4.** Mediation models of the effect of cumulative trauma up to age 15 on resting systolic blood pressure at age 18 through mediating substance use behaviours (problematic alcohol use, smoking, and illicit drug use; included individually)

|  | **Unadjusted** | **Adjusted** |
| --- | --- | --- |
| **Problematic alcohol use** | | |
| Total causal effect | -0·52 (-1·08, 0·05) | -0·59 (-1·11, -0·07) |
| Natural direct effect | -0·50 (-1·07, 0·08) | -0·54 (-1·06, -0·02) |
| Natural indirect effect | -0·02 (-0·08, 0·04) | -0·05 (-0·10, 0·003) |
| Proportion mediated | 3·8% | 8·6% |
| **Smoking** | | |
| Total causal effect | -0·53 (-1·09, 0·04) | -0·60 (-1·12, -0·08) |
| Natural direct effect | -0·46 (-1·03, 0·11) | -0·50 (-1·03, 0·03) |
| Natural indirect effect | -0·06 (-0·13, 0·004) | -0·10 (-0·17, -0·03) |
| Proportion mediated | 11·7% | 16·4% |
| **Illicit drug use** | | |
| Total causal effect | -0·52 (-1·08, 0·05) | -0·59 (-1·11, -0·07) |
| Natural direct effect | -0·51 (-1·08, 0·06) | -0·55 (-1·08, -0·03) |
| Natural indirect effect | -0·01 (-0·06, 0·04) | -0·04 (-0·09, 0·02) |
| Proportion mediated | 1·7% | 6·0% |

*Note*. Based on imputed data (*n*=3196). Baseline confounders include child sex, child ethnicity, maternal smoking during pregnancy, maternal alcohol consumption during pregnancy, maternal education at birth, monthly family income at birth, and cohort birth order (lower numbers correspond to birth earlier in the year). Intermediate confounders include age-adjusted BMI and estimated hours of physical activity at age 15.

**Table S5.** Mediation models of the effect of cumulative trauma up to age 15 on resting diastolic blood pressure at age 18 through mediating substance use behaviours (problematic alcohol use, smoking, and illicit drug use; included individually)

|  | **Unadjusted** | **Adjusted** |
| --- | --- | --- |
| **Problematic alcohol use** | | |
| Total causal effect | -0·72 (-1·06, -0·39) | -0·71 (-1·06, -0·36) |
| Natural direct effect | -0·66 (-1·00, -0·32) | -0·67 (-1·02, -0·31) |
| Natural indirect effect | -0·06 (-0·11, -0·02) | -0·04 (-0·08, -0·01) |
| Proportion mediated | 8·6% | 6·2% |
| **Smoking** | | |
| Total causal effect | -0·73 (-1·06, -0·39) | -0·72 (-1·07, -0·36) |
| Natural direct effect | -0·68 (-1·02, -0·34) | -0·66 (-1·02, -0·30) |
| Natural indirect effect | -0·05 (-0·09, -0·01) | -0·05 (-0·09, -0·02) |
| Proportion mediated | 6·5% | 7·5% |
| **Illicit drug use** | | |
| Total causal effect | -0·72 (-1·06, -0·39) | -0·71 (-1·06, -0·36) |
| Natural direct effect | -0·70 (-1·04, -0·37) | -0·70 (-1·06, -0·34) |
| Natural indirect effect | -0·02 (-0·06, -0·02) | -0·01 (-0·04, 0·01) |
| Proportion mediated | 2·4% | 1·5% |

*Note*. Based on imputed data (*n*=3196). Baseline confounders include child sex, child ethnicity, maternal smoking during pregnancy, maternal alcohol consumption during pregnancy, maternal education at birth, monthly family income at birth, and cohort birth order (lower numbers correspond to birth earlier in the year). Intermediate confounders include age-adjusted BMI and estimated hours of physical activity at age 15.

**Supplementary Complete Case Analyses**

**Descriptives**

Sample characteristics according to dichotomous trauma exposure status at age 18 are presented in Table S6 (p 10). By age 15, of 1918 adolescents with complete data, 30·4%, 20·9%, and 26·6% had been exposed to 1, 2, or ≥3 traumas in their lifetime. By age 18, of 3162 adolescents with complete data, 36·2%, 19·7%, and 25·2% had been exposed to 1, 2, or ≥3 traumas in their lifetime. Trauma-exposed adolescents were less likely to be white, and were more likely to be male, and their mothers had fewer years of education and were more likely to have smoked and consumed alcohol during pregnancy. At age 18, 30·5% of adolescents reported problematic alcohol use, 7·7% reported smoking, 24·3% reported illicit drug use, and the average sleep duration was 7 hours and 22 minutes. Descriptive statistics for adolescent risk behaviours and cardiovascular health indices according to cumulative trauma exposure up to age 15 are presented in Table S7 (p 11).

**Sex Differences**

Two interaction effects were found to be significant. The cross-sectional association between cumulative trauma and current illicit drug use at age 18 significantly differed by sex (interaction effect *p*-value=0·038). Sex-stratified analyses revealed that the OR for cumulative in males (adjusted OR=1·37 [95% CI 1·21-1·56], *p*<0·001) was greater than the OR for cumulative trauma in females (1·19 [1·05-1·35], *p*=0·006). Similarly, the longitudinal association between cumulative trauma up to age 11 and current illicit drug use at age 18 significantly differed by sex (interaction effect *p*-value=0·007). Sex-stratified analyses revealed that the OR for cumulative trauma in males (1·25 [1·09-1·45], *p*=0·002) was greater than the OR for cumulative trauma in females (0·94 [0·80-1·11], *p*=0·464).

**Population Attributable Fractions (PAFs)**

When treating trauma exposure up to age 18 as a binary variable (present/absent), PAFs (adjusted for baseline confounders) showed that trauma exposure up to age 18 accounted for 34·0% (95% CI 23·4-42·7) of problematic alcohol use, 52·2% (29·9-65·6) of current smoking, and 24·9% (12·3-35·1) of current illicit drug use at age 18.

**Table S6.** Sample characteristics according to trauma exposure status at age 18

|  | **Total Sample** | **Trauma at age 18** | | **OR (95% CI) or  Mean Difference** | ***p*-value** |
| --- | --- | --- | --- | --- | --- |
|  |  | **Unexposed (*n=*508)** | **Exposed (*n*=2066)** |  |  |
| **Binary variables** | | | | | |
| Child sex (female) | 1254 (48·7) | 269 (53·0) | 985 (47·7) | 0·81 (0·67, 0·98) | 0·033 |
| Child ethnicity (black/other)^a^ | 803 (31·2) | 121 (23·8) | 682 (33·0) | 1·58 (1·26, 1·97) | <0·001 |
| Maternal smoking (yes) | 676 (26·3) | 101 (19·9) | 575 (27·8) | 1·55 (1·22, 1·97) | <0·001 |
| Maternal alcohol consumption (yes) | 86 (3·3) | 9 (1·8) | 77 (3·7) | 2·15 (1·07, 4·31) | 0·032 |
| Problematic alcohol use at age 18 (yes) | 785 (30·5) | 96 (18·9) | 689 (33·4) | 2·15 (1·69, 2·73) | <0·001 |
| Smoking at age 18 (yes) | 199 (7·7) | 16 (3·2) | 183 (8·9) | 2·99 (1·78, 5·03) | <0·001 |
| Illicit drug use at age 18 (yes) | 626 (24·3) | 87 (17·1) | 539 (26·1) | 1·71 (1·33, 2·20) | <0·001 |
| **Continuous variables** | | | | | |
| Monthly family income, BRL^b^ | 803·05 (1040.24) | 866·49 (1008·83) | 787·45 (1047·46) | -79·05 (-180·04, 21·94) | 0·125 |
| Maternal education, years | 8·39 (3·33) | 8·68 (3·49) | 8·31 (3·29) | -0·37 (-0·69, -0·05) | 0·025 |
| Cohort birth order^c^ | 183·68 (103·38) | 206·18 (103·04) | 178·14 (102·74) | -28·03 (-38·01, -18·05) | <0·001 |
| Sleep duration at age 18 (hours) | 7·37 (1·38) | 7·41 (1·27) | 7·36 (1·40) | -0·05 (-0·18, 0·09) | 0·481 |

*Note*. Based on complete case data (*n*=2574). Data are *n* (%) for binary variables and mean (SD) for continuous variables, unless stated otherwise. ^a^Reference group is white. ^b^Conversion rate on January 1^st^, 2004: 1 BRL=0.34 USD. ^c^Ranked date of birth relative to other cohort members (lower numbers correspond to birth earlier in the year). *Key:* BRL=Brazilian Real. CI=confidence interval. OR=odds ratio.

**Table S7.** Descriptive statistics for adolescent risk behaviours and cardiovascular health indices according to cumulative trauma exposure up to age 15

|  | **Total Sample** | **Cumulative trauma exposure up to age 15** | | | |
| --- | --- | --- | --- | --- | --- |
|  |  | **0 traumas (*n*=230)** | **1 trauma (*n*=313)** | **2 traumas (*n*=225)** | **≥3 traumas (*n*=253)** |
| **Binary variables** | | | | | |
| Problematic alcohol use at age 18^a^ | 321 (31·4) | 51 (22·2) | 91 (29·1) | 79 (35·1) | 100 (39·5) |
| Current smoking at age 18 | 63 (6·2) | 7 (3·0) | 17 (5·4) | 15 (6·7) | 24 (9·5) |
| Current illicit drug use at age 18 | 231 (22·6) | 41 (17·8) | 61 (19·5) | 57 (25·3) | 72 (28·5) |
| **Continuous variables** | | | | | |
| Sleep duration at age 18 (hours) | 7·38 (1·36) | 7·45 (1·34) | 7·36 (1·31) | 7·42 (1·36) | 7·30 (1·43) |
| Resting HR at age 18 (bpm) | 78·07 (13·78) | 81·51 (15·01) | 77·92 (13·45) | 77·52 (13·59) | 75·61 (12·61) |
| Resting systolic BP at age 18 (mmHg) | 122·03 (13·25) | 122·18 (13·38) | 122·72 (13·51) | 122·69 (13·44) | 120·43 (12·55) |
| Resting diastolic BP at age 18 (mmHg) | 69·26 (8·10) | 70·45 (8·20) | 69·66 (8·39) | 68·99 (7·80) | 67·91 (7·76) |
| Physical activity at age 15 (hours) | 8·58 (10·05) | 7·74 (8·11) | 9·53 (11·72) | 8·38 (9·53) | 8·35 (9·82) |
| BMI at age 15 (age-adjusted z-score) | 0·49 (1·33) | 0·52 (1·34) | 0·40 (1·37) | 0·57 (1·33) | 0·50 (1·25) |

*Note.* Based on complete case data (*n*=1021). Data are *n* (%) for binary variables and mean (SD) for continuous variables. ^a^Based on the Alcohol Use Disorders Identification Test [(AUDIT);](https://assets.publishing.service.gov.uk/media/6357a7af8fa8f557d85b7c44/Alcohol-use-disorders-identification-test-AUDIT_for-print.pdf) adolescents scoring ≥8 were coded as having problematic alcohol use. *Key*: BP=diastolic blood pressure. HR=heart rate.

**Table S8.** Cross-sectional and longitudinal associations between cumulative trauma up to ages 11, 15, and 18 and adolescent risk behaviours at age 18

|  | **Cumulative trauma up to age 11 (*n*=2555)** | | **Cumulative trauma up to age 15 (*n*=1457)** | | **Cumulative trauma up to age 18 (*n*=2574)** | |
| --- | --- | --- | --- | --- | --- | --- |
|  | **Coefficient (95% CI)** | ***p*-value** | **Coefficient (95% CI)** | ***p*-value** | **Coefficient (95% CI)** | ***p*-value** |
| **Unadjusted** | | | | | | |
| Problematic alcohol use^a^ | 1·07 (0·97-1·19) | 0·172 | 1·32 (1·19-1·46) | <0·001 | 1·42 (1·31-1·54) | <0·001 |
| Current smoking | 1·18 (1·01-1·39) | 0·040 | 1·54 (1·26-1·87) | <0·001 | 1·56 (1·36-1·80) | <0·001 |
| Current illicit drug use | 1·13 (1·02-1·25) | 0·025 | 1·25 (1·12-1·40) | <0·001 | 1·30 (1·19-1·42) | <0·001 |
| Sleep duration (hours) | -0·006 (-0·07-0·06) | 0·853 | -0·05 (-0·11-0·02) | 0·148 | -0·03 (-0·08-0·02) | 0·244 |
| **Adjusted** | | | | | | |
| Problematic alcohol use^a^ | 1·03 (0·93-1·14) | 0·589 | 1·29 (1·16-1·43) | <0·001 | 1·38 (1·27-1·50) | <0·001 |
| Current smoking | 1·11 (0·94-1·31) | 0·208 | 1·49 (1·22-1·81) | <0·001 | 1·47 (1·27-1·70) | <0·001 |
| Current illicit drug use | 1·10 (0·99-1·22) | 0·088 | 1·23 (1·10-1·38) | <0·001 | 1·28 (1·17-1·39) | <0·001 |
| Sleep duration (hours) | -0·02 (-0·08-0·05) | 0·595 | -0·05 (-0·12-0·01) | 0·128 | -0·04 (-0·09-0·007) | 0·094 |

*Note*. Based on data from adolescents with complete data for trauma exposure, adolescent risk behaviours, and confounders. Coefficients for binary risk behaviours are odds ratios. Coefficients for continuous risk behaviours are unstandardised beta. Confounders include child sex, child ethnicity, maternal smoking during pregnancy, maternal alcohol consumption during pregnancy, maternal education at birth, monthly family income at birth, and cohort birth order. ^a^Based on the Alcohol Use Disorders Identification Test [(AUDIT);](https://assets.publishing.service.gov.uk/media/6357a7af8fa8f557d85b7c44/Alcohol-use-disorders-identification-test-AUDIT_for-print.pdf) adolescents scoring 8 and above were coded as having problematic alcohol use. *Key*: CI=confidence interval.

**Table S9.** Cross-sectional associations between adolescent risk behaviours at age 18 and resting HR, systolic BP, and diastolic BP at age 18

|  | **Problematic alcohol use** | | **Smoking** | | **Illicit drug use** | | **Sleep duration** | |
| --- | --- | --- | --- | --- | --- | --- | --- | --- |
|  | ***B* (95% CI)** | ***p*** | ***B* (95% CI)** | ***p*** | ***B* (95% CI)** | ***p*** | ***B* (95% CI)** | ***p*** |
| **Unadjusted** | | | | | | | | |
| HR | -2·66 (-4·48, -0·84) | 0·004 | 0·48 (-3·04, 4·00) | 0·790 | -4·30 (-6·31, -2·30) | <0·001 | 0·70 (0·08, 1·32) | 0·027 |
| Systolic BP | 0·99 (-0·76, 2·74) | 0·267 | -3·40 (-6·78, -0·03) | 0·048 | -1·07 (-3·01, 0·88) | 0·282 | -0·40 (-1·00, 0·20) | 0·190 |
| Diastolic BP | -0·91 (-1·98, 0·17) | 0·097 | -2·10 (-4·16, -0·03) | 0·046 | -1·62 (-2·81, -0·44) | 0·007 | 0·02 (-0·35, 0·39) | 0·921 |
| **Adjusted** | | | | | | | | |
| HR | -1·23 (-3·00, 0·54) | 0·172 | 1·06 (-2·35, 4·47) | 0·542 | -3·12 (-5·06, -1·18) | 0·002 | 0·29 (-0·31, 0·89) | 0·343 |
| Systolic BP | 0·84 (-0·71, 2·39) | 0·287 | -3·73 (-6·70, -0·77) | 0·014 | -1·06 (-2·76, 0·64) | 0·223 | 0·31 (-0·21, 0·84) | 0·245 |
| Diastolic BP | -0·41 (-1·46, 0·64) | 0·442 | -2·48 (-4·50, -0·46) | 0·016 | -1·25 (-2·40, -0·09) | 0·035 | -0·03 (-0·39, 0·33) | 0·876 |

*Note*. Based on data from adolescents with complete data for trauma exposure up to age 15, adolescent risk behaviours at age 18, resting HR, systolic BP, and diastolic BP at age 18, and baseline and intermediate confounders (*n*=1021). Confounders include child sex, child ethnicity, maternal smoking during pregnancy, maternal alcohol consumption during pregnancy, maternal education at birth, monthly family income at birth, cohort birth order, cumulative trauma up to age 15, age-adjusted BMI, and estimated hours of physical activity at age 15. *Key*: BP=blood pressure. CI=confidence interval. HR=heart rate.

**Table S10.** Mediation models of the effect of cumulative trauma up to age 15 on resting HR, systolic BP, and diastolic BP at age 18 through mediating substance use behaviours (problematic alcohol use, smoking, and illicit drug use; simultaneously included in the models)

|  | **Unadjusted** | **Adjusted** |
| --- | --- | --- |
| **Resting HR** | | |
| Total causal effect | -1·74 (-2·59, -0·89) | -1·61 (-2·30, -0·92) |
| Natural direct effect | -1·58 (-2·45, -0·72) | -1·50 (-2·15, -0·84) |
| Natural indirect effect | -0·16 (-0·35, 0·03) | -0·11 (-0·26, 0·04) |
| Proportion mediated | 9·2% | 6·9% |
| **Resting systolic BP** | | |
| Total causal effect | -0·58 (-1·18, 0·02) | -0·74 (-1·42, -0·06) |
| Natural direct effect | -0·57 (-1·17, 0·03) | -0·71 (-1·35, -0·07) |
| Natural indirect effect | -0·01 (-0·16, 0·15) | -0·02 (-0·19, 0·14) |
| Proportion mediated | 1·2% | 3·0% |
| **Resting diastolic BP** | | |
| Total causal effect | -0·83 (-1·31, -0·35) | -0·89 (-1·36, -0·41) |
| Natural direct effect | -0·75 (-1·21, -0·28) | -0·81 (-1·29, -0·34) |
| Natural indirect effect | -0·08 (-0·17, 0·004) | -0·08 (-0·16, 0·01) |
| Proportion mediated | 10·2% | 8·6% |

*Note*. Based on data from adolescents with complete data for all analysis variables (*n*=1021). Baseline confounders include child sex, child ethnicity, maternal smoking during pregnancy, maternal alcohol consumption during pregnancy, maternal education at birth, monthly family income at birth, and cohort birth order. Intermediate confounders include age-adjusted BMI and estimated hours of physical activity at age 15. *Key*: BP=blood pressure. HR=heart rate.

**Table S11.**  Mediation models of the effect of cumulative trauma up to age 15 on resting heart rate at age 18 through mediating substance use behaviours (problematic alcohol use, smoking, and illicit drug use; included individually)

|  | **Unadjusted** | **Adjusted** |
| --- | --- | --- |
| **Problematic alcohol use** | | |
| Total causal effect | -1·77 (-2·67, -0·88) | -1·57 (-2·36, -0·79) |
| Natural direct effect | -1·64 (-2·52, -0·75) | -1·53 (-2·32, -0·73) |
| Natural indirect effect | -0·14 (-0·25, -0·02) | -0·05 (-0·14, 0·05) |
| Proportion mediated | 7·6% | 3·0% |
| **Smoking** | | |
| Total causal effect | -1·75 (-2·65, -0·85) | -1·58 (-2·36, -0·79) |
| Natural direct effect | -1·78 (-2·67, -0·90) | -1·61 (-2·37, -0·84) |
| Natural indirect effect | 0·03 (-0·07, 0·13) | 0·03 (-0·04, 0·10) |
| Proportion mediated | -2·0% | -1·9% |
| **Illicit drug use** | | |
| Total causal effect | -1·78 (-2·67, -0·88) | -1·57 (-2·35, -0·79) |
| Natural direct effect | -1·61 (-2·50, -0·72) | -1·48 (-2·28, -0·69) |
| Natural indirect effect | -0·17 (-0·34, 0·005) | -0·09 (-0·20, 0·03) |
| Proportion mediated | 9·5% | 5·5% |

*Note*. Based on data from adolescents with complete data for all analysis variables (*n*=1021). Baseline confounders include child sex, child ethnicity, maternal smoking during pregnancy, maternal alcohol consumption during pregnancy, maternal education at birth, monthly family income at birth, and cohort birth order. Intermediate confounders include age-adjusted BMI and estimated hours of physical activity at age 15.

**Table S12.**  Mediation models of the effect of cumulative trauma up to age 15 on resting systolic blood pressure at age 18 through mediating substance use behaviours (problematic alcohol use, smoking, and illicit drug use; included individually)

|  | **Unadjusted** | **Adjusted** |
| --- | --- | --- |
| **Problematic alcohol use** | | |
| Total causal effect | -0·57 (-1·34, 0·19) | -0·71 (-1·36, -0·05) |
| Natural direct effect | -0·65 (-1·44, 0·14) | -0·73 (-1·39, -0·08) |
| Natural indirect effect | 0·08 (-0·02, 0·17) | 0·03 (-0·07, 0·12) |
| Proportion mediated | -13·2% | -3·8% |
| **Smoking** | | |
| Total causal effect | -0·59 (-1·36, 0·17) | -0·71 (-1·36, -0·06) |
| Natural direct effect | -0·52 (-1·29, 0·26) | -0·63 (-1·28, 0·03) |
| Natural indirect effect | -0·08 (-0·17, 0·02) | -0·09 (-0·19, 0·02) |
| Proportion mediated | 13·1% | 12·0% |
| **Illicit drug use** | | |
| Total causal effect | -0·59 (-1·35, 0·18) | -0·70 (-1·35, -0·05) |
| Natural direct effect | -0·55 (-1·32, 0·23) | -0·66 (-1·31, -0·002) |
| Natural indirect effect | -0·04 (-0·12, 0·04) | -0·04 (-0·12, 0·03) |
| Proportion mediated | 6·8% | 6·4% |

*Note*. Based on data from adolescents with complete data for all analysis variables (*n*=1021). Baseline confounders include child sex, child ethnicity, maternal smoking during pregnancy, maternal alcohol consumption during pregnancy, maternal education at birth, monthly family income at birth, and cohort birth order. Intermediate confounders include age-adjusted BMI and estimated hours of physical activity at age 15.

**Table S13.**  Mediation models of the effect of cumulative trauma up to age 15 on resting diastolic blood pressure at age 18 through mediating substance use behaviours (problematic alcohol use, smoking, and illicit drug use; included individually)

|  | **Unadjusted** | **Adjusted** |
| --- | --- | --- |
| **Problematic alcohol use** | | |
| Total causal effect | -0·83 (-1·28, -0·38) | -0·86 (-1·31, -0·41) |
| Natural direct effect | -0·79 (-1·25, -0·33) | -0·84 (-1·29, -0·38) |
| Natural indirect effect | -0·04 (-0·12, 0·04) | -0·02 (-0·09, 0·05) |
| Proportion mediated | 5·0% | 2·3% |
| **Smoking** | | |
| Total causal effect | -0·83 (-1·27, -0·39) | -0·86 (-1·31, -0·42) |
| Natural direct effect | -0·79 (-1·24, 0·33) | -0·81 (-1·27, -0·36) |
| Natural indirect effect | -0·04 (-0·09, 0·01) | -0·05 (-0·10, -0·002) |
| Proportion mediated | 5·2% | 5·6% |
| **Illicit drug use** | | |
| Total causal effect | -0·83 (-1·27, -0·39) | -0·86 (-1·30, -0·41) |
| Natural direct effect | -0·77 (-1·22, -0·32) | -0·82 (-1·27, -0·36) |
| Natural indirect effect | -0·06 (-0·15, 0·02) | -0·04 (-0·08, 0·005) |
| Proportion mediated | 7·4% | 4·5% |

*Note*. Based on data from adolescents with complete data for all analysis variables (*n*=1021). Baseline confounders include child sex, child ethnicity, maternal smoking during pregnancy, maternal alcohol consumption during pregnancy, maternal education at birth, monthly family income at birth, and cohort birth order. Intermediate confounders include age-adjusted BMI and estimated hours of physical activity at age 15.
