# Supplementary material for: Childhood trauma, adolescent risk behaviours and cardiovascular health indices in the 2004 Pelotas Birth Cohort
Source: J Child Psychol Psychiatry. 2025 Apr 30;66(11):1653–63. doi: 10.1111/jcpp.14173 (PMC12571934; doi:10.1111/jcpp.14173)
Supplement: Supplementary file 3 — Appendix S3. Brazilian Portuguese abstract translation. [file JCPP-66-1653-s002.docx]

**Exposição a trauma durante a infância, comportamentos de risco na adolescência e indicadores de saúde cardiovascular na Coorte de Nascimentos de Pelotas de 2004**

**RESUMO**

**Histórico**. O trauma na infância tem sido associado a um risco aumentado de uso de substâncias e sono de má qualidade, fatores esses que estão ligados a uma pior saúde cardiovascular posteriormente. No entanto, há pouca pesquisa longitudinal explorando essas associações na adolescência, especialmente em países de baixa e média renda. Para abordar essa lacuna, investigamos a associação entre trajetórias longitudinais do trauma e comportamentos de risco e indicadores de saúde cardiovascular entre adolescentes da Coorte de Nascimentos de Pelotas de 2004, no Brasil.

**Métodos.** O trauma cumulativo ao longo da vida foi avaliado por meio de relatos dos cuidadores até os 11 anos e, posteriormente, por relato combinado do cuidador e adolescente. Aos 18 anos, o uso problemático de álcool, o tabagismo, o uso de drogas ilícitas e a duração do sono foram medidos por auto-relato, e a frequência cardíaca e a pressão arterial em repouso foram avaliadas usando técnicas padronizadas. Testamos as associações entre trauma, comportamentos de risco e frequência cardíaca/pressão arterial utilizando regressão multivariada, frações atribuíveis populacionais e mediação contrafactual.

**Resultados.** Dos 4229 adolescentes (51·9% meninos), 81·9% haviam sido expostos a traumas até os 18 anos. O trauma acumulado até os 15 e até os 18 anos aumentou as chances de uso de álcool, tabagismo e de drogas aos 18 anos (OR ajustado: 1·25–1·44). A duração do sono não teve relação com o trauma na infância. As frações atribuíveis populacionais indicaram que o trauma infantil explicou ≥28% do uso de substâncias aos 18 anos. De forma inesperada, maior exposição ao trauma foi associada a menor frequência cardíaca e pressão arterial em repouso. O uso de substâncias mediou parcialmente o efeito do trauma sobre os indicadores de saúde cardiovascular.

**Interpretação.** O trauma está associado ao uso de substâncias entre adolescentes em países de baixa e média renda. Estratégias de prevenção e intervenção focadas no trauma são essenciais, considerando esse impacto significativo. Nossa descoberta de que o trauma prediz menor frequência cardíaca e pressão arterial merece investigação adicional, dada a associação bem estabelecida entre trauma e pior saúde cardiovascular na idade adulta.
