# Supplementary material for: Childhood trauma, adolescent risk behaviours and cardiovascular health indices in the 2004 Pelotas Birth Cohort
Source: J Child Psychol Psychiatry. 2025 Apr 30;66(11):1653–63. doi: 10.1111/jcpp.14173 (PMC12571934; doi:10.1111/jcpp.14173)
Supplement: Supplementary file 4 — Appendix S4. STROBE statement—checklist of items that should be included in reports of observational studies. [file JCPP-66-1653-s003.docx]

STROBE Statement—checklist of items that should be included in reports of observational studies

|  | Item No. | Recommendation | Page  No. | Relevant text from manuscript |
| --- | --- | --- | --- | --- |
| **Title and abstract** | 1 | (*a*) Indicate the study’s design with a commonly used term in the title or the abstract | 1-2 | 2004 Pelotas Birth Cohort |
|  |  | (*b*) Provide in the abstract an informative and balanced summary of what was done and what was found | 2 | See Abstract |
| Introduction | | | |  |
| Background/rationale | 2 | Explain the scientific background and rationale for the investigation being reported | 4-5 | See Introduction |
| Objectives | 3 | State specific objectives, including any prespecified hypotheses | 5 | See Introduction |
| Methods | | | |  |
| Study design | 4 | Present key elements of study design early in the paper | 6 | See Methods: ‘Study design and population’ subsection |
| Setting | 5 | Describe the setting, locations, and relevant dates, including periods of recruitment, exposure, follow-up, and data collection | 6 | See Methods: ‘Study design and population’ subsection |
| Participants | 6 | (*a*) *Cohort study*—Give the eligibility criteria, and the sources and methods of selection of participants. Describe methods of follow-up  *Case-control study*—Give the eligibility criteria, and the sources and methods of case ascertainment and control selection. Give the rationale for the choice of cases and controls  *Cross-sectional study*—Give the eligibility criteria, and the sources and methods of selection of participants | 6 | See Methods: “*All hospitals with maternity wards were visited daily, and all live births were eligible for enrolment. Children were assessed at birth, 3, 12, 24, and 48 months, then at 6, 11, 15, and 18 years*.” |
|  |  | (*b*) *Cohort study*—For matched studies, give matching criteria and number of exposed and unexposed  *Case-control study*—For matched studies, give matching criteria and the number of controls per case | 10 | See Results: “*By age 11, 21·7%, 7·2%, and 5·3% of the cohort had been exposed to 1, 2, or ≥3 traumas in their lifetime, respectively. By age 15, 30·1%, 20·8%, and 27·2% of adolescents had been exposed to 1, 2, or ≥3 traumas in their lifetime, respectively. By age 18, 36·2%, 19·8%, and 25·3% of adolescents had been exposed to 1, 2, or ≥3 traumas in their lifetime, respectively.*” |
| Variables | 7 | Clearly define all outcomes, exposures, predictors, potential confounders, and effect modifiers. Give diagnostic criteria, if applicable | 6-8 | See Methods: ‘Measures’ subsection |
| Data sources/ measurement | 8* | For each variable of interest, give sources of data and details of methods of assessment (measurement). Describe comparability of assessment methods if there is more than one group | 6-8 | See Methods: ‘Measures’ subsection |
| Bias | 9 | Describe any efforts to address potential sources of bias | 8 | See Methods: ‘Data analysis’ subsection – “*Multivariate imputation by chained equations with 50 imputed datasets was used to address missing data (for further details see Appendix 1, p 5-6). Findings presented here are based on imputed data (for complete case analyses see Appendix 2, p 9-17).*” |
| Study size | 10 | Explain how the study size was arrived at | 8 | See Methods: ‘Data analysis’ subsection – “*Next, due to an absence of feasible auxiliary variables for the imputation of resting HR at age 18, our mediation analyses used imputed data for a subsample of the cohort who had complete resting HR data (n=3196)*.” |

| Quantitative variables | 11 | Explain how quantitative variables were handled in the analyses. If applicable, describe which groupings were chosen and why | 6-8 | See Methods: ‘Measures’ subsection |
| --- | --- | --- | --- | --- |
| Statistical methods | 12 | (*a*) Describe all statistical methods, including those used to control for confounding | 8-9 | See Methods: ‘Data analysis’ subsection |
|  |  | (*b*) Describe any methods used to examine subgroups and interactions | 8 | See Methods: ‘Data analysis’ subsection – “*In sensitivity analyses, we examined whether associations differed by sex*.” |
|  |  | (*c*) Explain how missing data were addressed | 8 | See Methods: ‘Data analysis’ subsection – “*Multivariate imputation by chained equations with 50 imputed datasets was used to address missing data (for further details see Appendix 1, p 5-6). Findings presented here are based on imputed data (for complete case analyses see Appendix 2, p 9-17).*” |
|  |  | (*d*) *Cohort study*—If applicable, explain how loss to follow-up was addressed  *Case-control study*—If applicable, explain how matching of cases and controls was addressed  *Cross-sectional study*—If applicable, describe analytical methods taking account of sampling strategy | 8 | See Methods: ‘Data analysis’ subsection – “*Multivariate imputation by chained equations with 50 imputed datasets was used to address missing data (for further details see Appendix 1, p 5-6). Findings presented here are based on imputed data (for complete case analyses see Appendix 2, p 9-17).*” |
|  |  | (*e*) Describe any sensitivity analyses | 8-9 | See Methods: ‘Data analysis’ subsection |
| Results | | | | |
| Participants | 13* | (a) Report numbers of individuals at each stage of study—eg numbers potentially eligible, examined for eligibility, confirmed eligible, included in the study, completing follow-up, and analysed | - | See Supplement 1, p 2: ‘Cohort Details’ subsection |
|  |  | (b) Give reasons for non-participation at each stage | - | See Supplement 1, p 2: ‘Cohort Details’ subsection |
|  |  | (c) Consider use of a flow diagram | - | Flow diagrams for the cohort have previously been presented in cohort profiles. |
| Descriptive data | 14* | (a) Give characteristics of study participants (eg demographic, clinical, social) and information on exposures and potential confounders | 30 | See Table 1 |
|  |  | (b) Indicate number of participants with missing data for each variable of interest | - | See Supplement 1, Table S2 |
|  |  | (c) *Cohort study*—Summarise follow-up time (eg, average and total amount) | - | See Supplement 1, p 2: ‘Cohort Details’ subsection |
| Outcome data | 15* | *Cohort study*—Report numbers of outcome events or summary measures over time | 10 | See Results: “*At age 18, 30·3% of adolescents reported problematic alcohol use, 8·6% reported smoking, 27·1% reported illicit drug use, and average sleep duration was 7 hours and 26 minutes*.” |
|  |  | *Case-control study—*Report numbers in each exposure category, or summary measures of exposure |  |  |
|  |  | *Cross-sectional study—*Report numbers of outcome events or summary measures |  |  |
| Main results | 16 | (*a*) Give unadjusted estimates and, if applicable, confounder-adjusted estimates and their precision (eg, 95% confidence interval). Make clear which confounders were adjusted for and why they were included | 8 | See Methods: ‘Data analysis’ subsection – “*All analyses were conducted twice: unadjusted and adjusted for baseline confounders.*” Further details regarding the choice of confounders is presented in Supplement 1 (p 4). Unadjusted and adjusted estimates are presented in all tables. |
|  |  | (*b*) Report category boundaries when continuous variables were categorized | 6 | See Methods: ‘Measures’ subsection – “*The resultant variables were coded as 0, 1, 2, and ≥3 exposures*.” |
|  |  | (*c*) If relevant, consider translating estimates of relative risk into absolute risk for a meaningful time period | n/a | n/a |

| Other analyses | 17 | Report other analyses done—eg analyses of subgroups and interactions, and sensitivity analyses | 10-13 | See Results |
| --- | --- | --- | --- | --- |
| Discussion | | | | |
| Key results | 18 | Summarise key results with reference to study objectives | 13-14 | See Discussion |
| Limitations | 19 | Discuss limitations of the study, taking into account sources of potential bias or imprecision. Discuss both direction and magnitude of any potential bias | 18-19 | See Discussion |
| Interpretation | 20 | Give a cautious overall interpretation of results considering objectives, limitations, multiplicity of analyses, results from similar studies, and other relevant evidence | 19-20 | See Conclusion |
| Generalisability | 21 | Discuss the generalisability (external validity) of the study results | 19 | See Discussion: “*Finally, our findings should be interpreted in the context of Pelotas, a predominantly urban area with a higher income inequality and lower gross domestic product per capita compared to the Brazilian national average (see Supplement 1, p 2); the generalizability of our findings may therefore be limited given the considerable socioeconomic variation in Brazil.*” |
| Other information | |  | | |
| Funding | 22 | Give the source of funding and the role of the funders for the present study and, if applicable, for the original study on which the present article is based | 21 | See Acknowledgements |

*Give information separately for cases and controls in case-control studies and, if applicable, for exposed and unexposed groups in cohort and cross-sectional studies.

**Note:** An Explanation and Elaboration article discusses each checklist item and gives methodological background and published examples of transparent reporting. The STROBE checklist is best used in conjunction with this article (freely available on the Web sites of PLoS Medicine at http://www.plosmedicine.org/, Annals of Internal Medicine at http://www.annals.org/, and Epidemiology at http://www.epidem.com/). Information on the STROBE Initiative is available at www.strobe-statement.org.
